# Lipid-driven CFTR clustering is impaired in cystic fibrosis and restored by corrector drugs

**DOI:** 10.1242/jcs.259002

**Published:** 2022-03-07

**Authors:** Asmahan Abu-Arish, Elvis Pandžić, Yishan Luo, Yukiko Sato, Mark J. Turner, Paul W. Wiseman, John W. Hanrahan

**Affiliations:** 1 Department of Physiology, McGill University, Montréal, QC H3G 1Y6, Canada; 2 Cystic Fibrosis Translational Research Centre, McGill University, Montréal, QC H3G 1Y6, Canada; 3 UNSW Australia, Biomedical Imaging Facility, Mark Wainwright Analytical Center, Sydney 2052, Australia; 4 Department of Physics, McGill University, Montréal, QC H3A 2T8, Canada; 5 Department of Chemistry, McGill University, Montréal, QC H3A 0B8, Canada; 6 Research Institute of the McGill University Health Centre, McGill University, Montréal, QC H3H 2R9, Canada

**Keywords:** Cystic fibrosis, PDZ-motif, Trikafta, Cholesterol, Ceramide, Filamin A

## Abstract

Membrane proteins often cluster in nanoscale membrane domains (lipid rafts) that coalesce into ceramide-rich platforms during cell stress, however the clustering mechanisms remain uncertain. The cystic fibrosis transmembrane conductance regulator (CFTR), which is mutated in cystic fibrosis (CF), forms clusters that are cholesterol dependent and become incorporated into long-lived platforms during hormonal stimulation. We report here that clustering does not involve known tethering interactions of CFTR with PDZ domain proteins, filamin A or the actin cytoskeleton. It also does not require CFTR palmitoylation but is critically dependent on membrane lipid order and is induced by detergents that increase the phase separation of membrane lipids. Clustering and integration of CFTR into ceramide-rich platforms are abolished by the disease mutations F508del and S13F and rescued by the CFTR modulators elexacaftor plus tezacaftor. These results indicate CF therapeutics that correct mutant protein folding restore both trafficking and normal lipid interactions in the plasma membrane.

This article has an associated First Person interview with the first author of the paper.

## Abstract (precis)

**Summary:** CFTR clustering in ceramide-rich platforms is independent of protein–protein interactions, driven by lipid phase separation, prevented by CFTR mutations that cause misfolding and restored by Trikafta correctors.

## INTRODUCTION

The cystic fibrosis transmembrane conductance regulator (CFTR) anion channel plays important roles in airway physiology and host defense. Loss-of-function mutations in CFTR cause cystic fibrosis (CF), a multi-organ disease of chronic inflammation, mucus obstruction and bacterial infection that leads to a gradual decline in lung function (Ratjen et al., 2015). Deletion of phenylalanine at amino acid position 508 (F508del) is by far the most frequent CFTR mutation, occurring on at least one chromosome in ∼90% of CF patients. It causes misfolding, defective trafficking of the protein from the endoplasmic reticulum (ER) to the plasma membrane (PM) and premature degradation (Cheng et al., 1990). When F508del-CFTR escapes ER quality control and reaches the PM, its open probability is reduced compared to wild-type CFTR and it is internalized more rapidly, which may place a ceiling on the efficacy of CF therapeutics, several of which [lumacaftor (VX-809), elexacaftor (VX-445) and tezacaftor (VX-661)] are used clinically (Van Goor et al., 2011; Keating et al., 2018). Stabilizing CFTR mutants at the cell surface after pharmacological rescue is an important goal of CF research and strategies that are successful for CFTR mutants may be applicable to other protein folding diseases.

Primary human bronchial epithelial (HBE) cells have at least two CFTR populations on their surface under normal conditions, one that is localized in clusters or adjacent to cell–cell junctions, and another that is diffusely distributed (Abu-Arish et al., 2015). Clustered CFTR channels have relatively slow, confined movements over a small spatial scale, consistent with their localization in lipid microdomains, whereas the diffusely distributed membrane population has transport dynamics on larger spatial scales that reflect CFTR movements both inside and outside microdomains. The dependence of CFTR clustering on cholesterol implies that the clusters reside in dynamic, nanoscale liquid-ordered (L_o_) domains or lipid rafts that contain cholesterol and are enriched in sphingomyelins and gangliosides, such as GM1. Rafts merge to form large ceramide-rich platforms in response to pathological stimuli (Grassme et al., 2003) and physiological agonists that stimulate Cl^−^ and fluid secretion, such as the peptide hormone vasoactive intestinal peptide (VIP) and muscarinic agonist carbachol (Abu-Arish et al., 2019). Fusion of lipid rafts into long-lived ceramide platforms slows the rate of CFTR endocytosis thereby increasing CFTR functional expression by several fold during VIP stimulation (Abu-Arish et al., 2019). The formation of platforms and their role in regulating CFTR surface expression during physiological regulation were discovered only recently, perhaps because they are reduced or absent in cell lines that are commonly used in CF research.

Most CFTR is localized in the PM or in recycling endosomes in well-differentiated primary airway epithelial cells and its biosynthetic arrest in CF is associated with remarkable changes in the abundance and intracellular distribution of cholesterol and other lipids (Scholte et al., 2019; Garic et al., 2019; Freedman et al., 1999). The lipid interactome of CFTR has not been characterized; however, most cellular sphingomyelin and cholesterol (i.e. ∼90%) are situated in the PM, where the cholesterol may reach very high concentrations. Cholesterol exists in two forms, one that is deemed inaccessible due to binding to sphingomyelin and other membrane components, and a smaller ‘free’ cholesterol fraction that is available for non-vesicular transport to the ER by lipid transfer proteins at sites of PM–ER contact (Jeyasimman and Saheki, 2020). Free cholesterol is elevated in the PM and perinuclear compartments in CFTR-deficient cells and these changes are accompanied by increased cholesterol synthesis (White et al., 2004, 2007). Cholesterol levels are also increased in the late endosomes of cells that express F508del-CFTR, though not in those lacking CFTR completely, suggesting this abnormality results from the presence of the mutant protein rather than the loss of channel function (Fang et al., 2010; Gentzsch et al., 2007). CF cells have many lipid abnormalities including reduced levels of the polyunsaturated fatty acid dihydroarachidonic acid (DHA; 22:5ω3), elevated arachidonic acid (20:4ω6) (Freedman et al., 2004, 1999), and an increase in the ratio of long chain (e.g. C16:0, C18:0) to very long chain (e.g. C24:0, C24:1) ceramides (Garić et al., 2017; Veltman et al., 2021). Understanding the reciprocal interactions between CFTR and membrane lipids is important as membrane microdomains are implicated in the response to infection and airways host defense (Grassme et al., 2003; Aureli et al., 2016).

Here, we investigate the mechanism of clustering, beginning with the possible role of well-established CFTR interactions with PDZ domain proteins, filamin A and actin. We also examined the possible role of palmitoylation at C1395 of CFTR, as palmitoylation is the most common post-translational modification for targeting proteins to lipid rafts. The results show that CFTR clustering and recruitment into platforms are independent of scaffold proteins and are driven by lipid order, prevented by the disease-causing mutations F508del and S13F, and largely restored by corrector drugs that are currently used to treat CF.

## RESULTS

### CFTR clusters colocalize with fluorescent analogs of cholesterol and sphingomyelin

**Fig. 1.**
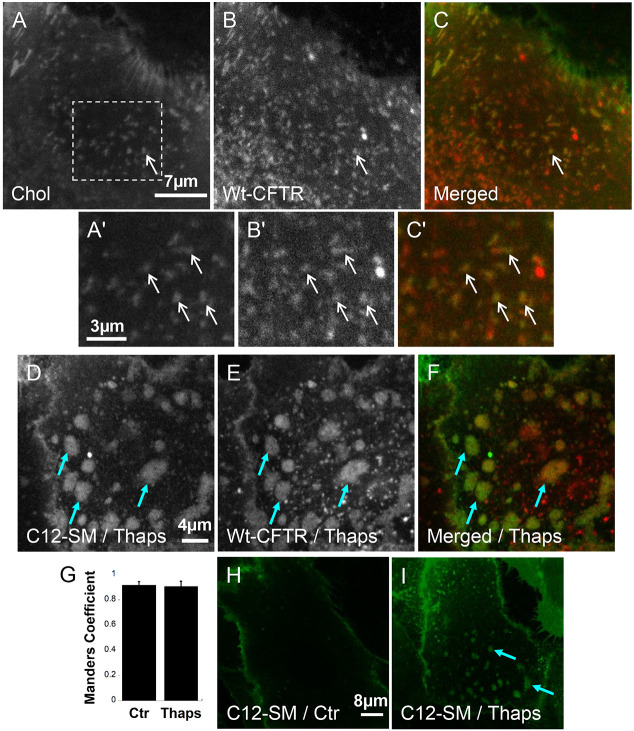
**CFTR clusters are associated with lipid rafts.** HBE cells were transduced with adenovirus containing mCherry–wt-CFTR. (A–C) Cells were loaded with BODIPY-cholesterol (Chol, 1 µM) complexed with methyl-β-cyclodextrin. Note colocalization of cholesterol with CFTR clusters (arrow). (A′–C′) Magnified view of the area indicated in A showing CFTR and cholesterol colocalization (*N*_exp_=4; *n*_cell_=102) (arrows). (D–F) Cells preloaded with BODIPY-C12-Sphingomyelin (C12-SM, 0.5 µg/ml) and stimulated with thapsigargin (Thaps) show localization of C12-SM in CFTR-rich platforms, suggesting C12-SM is hydrolyzed into C12-Ceramide (*N*_exp_=4; *n*_cell_=109). (G) Mander's overlap coefficient showing 90% spatial colocalization of CFTR and cholesterol inside clusters, and CFTR and C12-SM inside platforms. Results are mean±s.e.m. (*N*_exp_=2, *n*_ctr_= 20; *N*_exp_=3, *n*_thaps_=32). (H,I) Cells pre-loaded with C12-SM for 30 min failed to form platforms under Ctr conditions, and only Thaps treatment triggered their formation, suggesting that C12-SM treatment alone does not trigger platform formation. Blue arrows in D–F,I highlight ceramide-rich platform formation and CFTR localization therein.

To visualize the association of CFTR with membrane microdomains, we transduced primary HBE cells with an adenovirus containing wild-type CFTR with mCherry fused to its N-terminus (mCherry–wt-CFTR) and exposed the cells to fluorescent analogs of cholesterol and sphingomyelin using methyl-β-cyclodextrin (MβCD) as a lipid carrier. CFTR clusters were observed on the cell surface as described previously using EGFP–CFTR (Abu-Arish et al., 2015, 2019), and became associated with BODIPY-cholesterol within 2–3 min after addition (Fig. 1A–C, white arrow; highlighted in Fig. 1A′–C′). Raising intracellular Ca^2+^ with thapsigargin (Thaps, 2 µM), a specific inhibitor of the sarco/endoplasmic reticulum Ca^2+^-ATPase (SERCA) pump (Lytton et al., 1991), caused CFTR clusters to merge into large platforms within 10–15 min (Fig. 1E, blue arrows). We showed previously that these platforms, which resemble those formed during infection and cell stress (Grassme et al., 2003), arise when CFTR-containing lipid rafts coalesce into larger microdomains. Their formation is prevented by amitriptyline, which inhibits acid sphingomyelinase (ASMase) indirectly through release and degradation in lysosomes and also by direct inhibition of the enzyme (Abu-Arish et al., 2019; Gorelik, 2017). Since Ca^2+^ mobilization stimulates the hydrolysis of sphingomyelin to ceramide, we examined CFTR in cells that had been preloaded with BODIPY-C12-sphingomyelin (C12-SM). Thaps caused large-scale aggregation of both the BODIPY (i.e. BODIPY-C12-ceramide; Fig. 1D) and mCherry–wt-CFTR signals (Fig. 1E) into platforms 2–4 µm in diameter (Fig. 1F, blue arrows). Manders’ overlap coefficient analysis showed 90% spatial colocalization between CFTR and cholesterol inside clusters (Fig. 1G, Ctr), as well as CFTR and C12-ceramide inside platforms following Ca^2+^ mobilization with Thaps (Fig. 1G, Thaps). To exclude the possibility that C12-SM alone triggers platform formation, HBE cells were loaded with BODIPY-C12-SM for 30 min before imaging, and no platform formation was detected (Fig. 1H). Platforms only formed after treatment with Thaps (Fig. 1I). These results demonstrate that CFTR clusters associate with cholesterol- and sphingomyelin-containing lipid rafts, consistent with their sensitivity to cholesterol oxidase and esterase (Abu-Arish et al., 2015), and become incorporated into large platforms when intracellular Ca^2+^ is elevated.

### Clustering does not require tethering by PDZ domain proteins, filamin A or the actin cytoskeleton

**Fig. 2.**
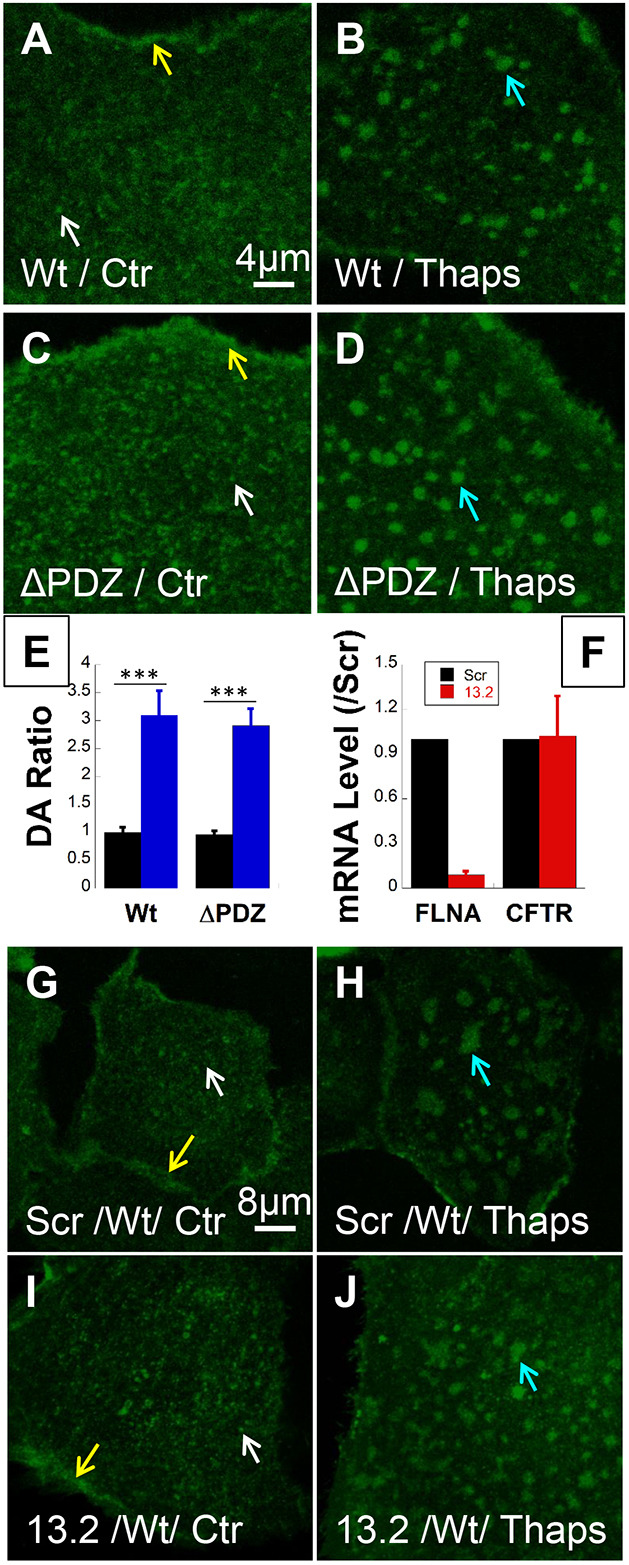
**CFTR clustering is independent of PDZ domain proteins and FLNA.** Confocal images of primary HBE cells transduced with adenoviruses that contain EGFP–wt-CFTR or EGFP–ΔPDZ-CFTR (called wt- or ΔPDZ-CFTR henceforth) after 4–5 days culture on collagen-coated glass. (A) PM distribution of wt-CFTR under control (Ctr, *N*_exp_=54; *n*_cell_=1099) conditions showing perijunctional localization (yellow arrow) and clusters (white arrow). (B) Thaps treatment causes aggregation of wt-CFTR clusters into large platforms (blue arrow; *N*_exp_=29; *n*_cell_=861). (C,D) Membrane distribution of CFTR truncation mutant lacking the PDZ-binding motif (ΔPDZ-CFTR) is similar to wt-CFTR under (C) Ctr conditions (*N*_exp_=14; *n*_cell_=421) and (D) after Thaps treatment (*N*_exp_=5; *n*_cell_=100). (E) Image correlation spectroscopy analysis showing the normalized degree of aggregation (DA ratio) of wt-CFTR and ΔPDZ-CFTR is similar. Thaps increases DA ratio by 3-fold indicating formation of higher-order aggregates (platforms). (mean±s.e.m.; *N*_exp_=2, *n*_cell_: *n*_wt_=59, *n*_wt+Thaps_=30, *n*_ΔPDZ_=130, *n*_ΔPDZ+Thaps_=25). ***P<0.0025 (unpaired one-tailed *t*-test). Each cell is an independent biological sample. (F) Relative mRNA levels of endogenous FLNA and overexpressed wt-CFTR after FLNA knockdown with dicer RNA duplexes (DsiRNA, RNA sequence 13.2) (mean±s.e.m.; *N*_exp_=4, *N*_patient_=3). Scr, scrambled DsiRNA. (G,H) Control cells transfected with scrambled RNA sequence had normal wt-CFTR clustering and peri-junctional localization (*N*_exp_=4; *n*_cell_=72) and formed platforms during Ca^2+^ stimulation by Thaps (*N*_exp_=4; *n*_cell_=69). (I,J) Normal wt-CFTR cluster (*N*_exp_=6; *n*_cell_=113) and platform formation (*N*_exp_=5; *n*_cell_= 89) after FLNA transcripts knockdown. Arrows in C,D,G–J are as for A,B.

When primary HBE cells were transduced with adenoviral CFTR bearing N-terminal EGFP (EGFP–wt-CFTR) we observed bright clusters (Fig. 2A, white arrow), diffusely distributed CFTR and a population that was localized near cell–cell junctions (Fig. 2A, yellow arrow, visible only in the transfected cell) as was reported previously (Abu-Arish et al., 2015). The channels were mainly localized in the PM (Fig. S1A). Thapsigargin caused CFTR clusters to fuse into large (2–4 µm) platforms within 10–15 min (Fig. 2B, blue arrow). CFTR has a C-terminal motif (DTRL) that interacts with multiple PDZ domain proteins that influence its trafficking (Cheng et al., 2002), endosome recycling (Swiatecka-Urban et al., 2002) and retrieval from the cell surface (Arora et al., 2014). To test whether a PDZ domain interaction controls CFTR clustering in lipid rafts, we compared the behavior of wt-CFTR (Fig. 2A,B) with a mutant lacking this motif (ΔPDZ-CFTR). The mutant formed normal clusters and was incorporated into platforms during Ca^2+^ stimulation like wt-CFTR (Fig. 2C,D). Moreover, the degree of aggregation measured using spatial image correlation spectroscopy analysis (DA ratio, an indicator of cluster size in terms of average number of CFTR molecules) was similar for the mutant and wt-CFTR under both control conditions (black bar) and during Thaps stimulation (Fig. 2E, blue bar) (Wiseman and Petersen, 1999; Abu-Arish et al., 2015). These results indicate that interactions with NHERF1 and NHERF2 (also known as SLC9A3R1 and SLC9A3R2, respectively), and other PDZ domain proteins are not required for CFTR clustering or for integration of the clusters into ceramide-rich platforms.

Filamin A (FLNA) is an actin-binding protein that mediates clustering of the glycoprotein CD4 (Jimenez-Baranda et al., 2007) and the type 2 somatostatin receptor (Treppiedi et al., 2018). It has multiple binding sites for CFTR (Smith et al., 2010), and restricts its lateral mobility in the PM of BHK cells (Thelin et al., 2007). To examine the possible role of FLNA in CFTR clustering, we used RNA interference [chemically synthesized Dicer substrate siRNA duplexes (13.2), IDT, Inc., Coralville, IA] to reduce the number of FLNA mRNA transcripts by >90% without altering CFTR mRNA expression (Fig. 2F) (Ramachandran et al., 2013). Cells transfected with a scrambled RNA sequence had normal peri-junctional localization (Fig. 2G, white and yellow arrows, respectively) and formed normal CFTR clusters and platforms during Ca^2+^ stimulation as expected (Fig. 2H). Identical results were obtained in FLNA-deficient cells (Fig. 2I,J). Immunostaining confirmed 80% knockdown of FLNA at the protein level (Fig. S2A–C), and in control cells there was no colocalization of FLNA with CFTR (Fig. S2). Taken together, these results indicate that FLNA does not mediate CFTR clustering or its incorporation into platforms during stimulation.

**Fig. 3.**
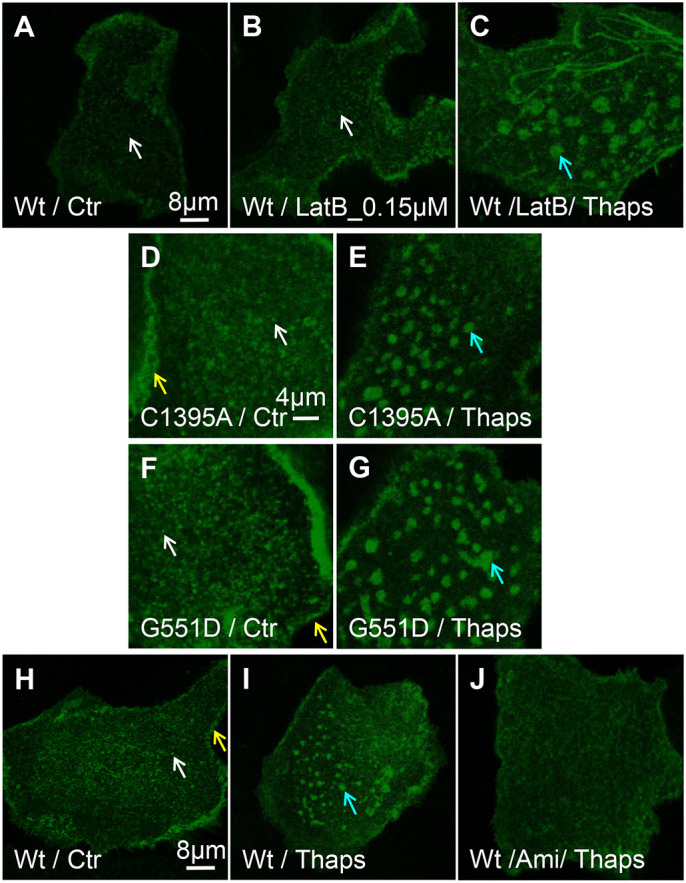
**Intact actin cytoskeleton, palmitoylation and channel gating are not required for wt-CFTR clustering or entry into platforms.** HBE cells were transduced with adenoviruses containing wt-, C1395A- or G551D-CFTR. (A,B) There is a similar distribution of wt-CFTR under Ctr conditions (*N*_exp_=12; *n*_cell_=260) and after treatment with the actin depolymerization reagent LatB (0.15 µM, *N*_exp_=8; *n*_cell_=314). (C) wt-CFTR entry into platforms after Thaps (*N*_exp_=8; *n*_cell_=132) was not affected by LatB (*N*_exp_=3; *n*_cell_=44), indicating the actin cytoskeleton does not influence CFTR cluster or platform formation at the PM. (D,E) Lack of palmitoylation at site 1395 (C1395A) does not hinder CFTR clustering under Ctr conditions (*N*_exp_=4; *n*_cell_=114) or aggregation into large platforms after Thaps treatment (*N*_exp_=3; *n*_cell_=69). (F,G) Distribution of G551D-CFTR, a membrane-stable mutant with defective gating is similar to wt-CFTR under all conditions (Ctr, *N*_exp_=15; *n*_cell_=329, Thaps, *N*_exp_=8; *n*_cell_=204). (H–J) Distribution of wt-CFTR under Ctr conditions shows cluster formation at the PM (*N*_exp_=12; *n*_cell_=260). (I) Coalescence of wt-CFTR clusters into large (2–4 µm dia) platforms after 10–20 min exposure to 2 µM thapsigargin (Thaps, *N*_exp_=8; *n*_cell_=132). (J) Pretreating cells with the acid sphingomyelinase inhibitor amitriptyline (Ami) inhibits ceramide generation, platform formation and wt-CFTR redistribution into them in response to Thaps (*N*_exp_=4; *n*_cell_=98). These results indicate that platform formation is driven by ceramide generation. White arrows highlight CFTR clustering, yellow arrows highlight CFTR perijunctional localization, and blue arrows highlight ceramide-rich platform formation and CFTR localization therein.

Actin interacts with CFTR and may modulate its activation by PKA (Prat et al., 1995; Fischer et al., 1995). We tested its role by transducing HBE cells using adenoviral EGFP–wt-CFTR and treating them with the actin-depolymerizing agent latrunculin B (LatB, 0.15 µM or 0.3 µM). Some cells were subsequently treated with Thaps (after LatB) to determine whether CFTR entry or retention in ceramide-rich platforms requires the actin cytoskeleton. Cells were then stained with phalloidin, fixed and imaged while focusing on the cell surface (Fig. S3). LatB did not affect CFTR clustering (compare Fig. 3A,B and Fig. S3D,G,J) or incorporation into platforms (Fig. 3C). Disruption of the cytoskeleton was confirmed by phalloidin staining after 0.15 and 0.3 µM LatB (Fig. S3B,C,H,K). The distribution of CFTR clusters was not correlated with residual actin filaments remaining after LatB treatment or with punctate actin staining (Fig. S3F,I,L). Similar results were obtained when cytochalasin D (CytoD, 0.5 µM) was used to disrupt the cytoskeleton (Fig. S4A–C), further indicating that the actin cytoskeleton is not required for CFTR clustering or incorporation into platforms.

### CFTR is not targeted to microdomains by C1395-palmitoylation

Palmitoylation is the most common post-translational modification for targeting proteins to lipid rafts (Levental et al., 2010) and has been demonstrated on CFTR at C1395 by liquid chromatography tandem mass spectrometry (LC-MS/MS) (McClure et al., 2012). A second palmitoylation site was also detected at C524 in the first nucleotide-binding domain using the more sensitive method of multiple reaction ion monitoring MS. We examined the impact of preventing palmitoylation at C1395, which is near the C-terminus where it could potentially reach the membrane inner leaflet, unlike C524 in NBD1 (distance ∼60 Å, palmitate length=21.9 Å; 1 Å=0.1 nm). Replacing cysteine 1395 with alanine (i.e. a C1395A-CFTR mutant) did not alter clustering or incorporation into platforms (Fig. 3D,E). Furthermore, inhibiting CFTR palmitoylation using the general palmitoylation inhibitor 2-bromo-palmitoyl (2BP, 10 µM for 24 h) did not alter CFTR cluster formation at the PM (Fig. S4D,E), further supporting the conclusion that palmitoylation does not target CFTR to lipid microdomains.

### The gating mutant G551D-CFTR forms normal clusters

We studied the third most common CF mutation, G551D, which folds and traffics to the cell surface normally but has impaired activation by PKA (Yang et al., 1993). The behavior of EGFP–G551D-CFTR was indistinguishable from that of wt-CFTR, localizing in clusters and near cell–cell junctions (Fig. 3F, white and yellow arrow, respectively), and coalescing into platforms during Thaps stimulation (Fig. 3G, blue arrow). Pre-treating cells with the acid sphingomyelinase inhibitor amitriptyline (Ami) prevented the appearance of large CFTR platforms (Fig. 3J), consistent with hydrolysis of raft sphingomyelin to ceramide and fusion of the rafts into larger domains. These results indicate that diminished Cl^−^ conductance and secondary changes in the lipid composition of CF cells do not affect CFTR clusters or their incorporation into platforms.

### CFTR clustering can be induced using detergents

**Fig. 4.**
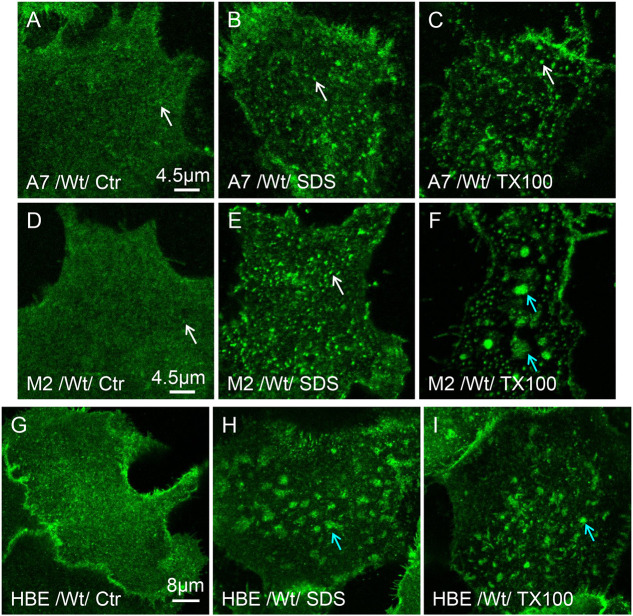
**Detergents enhance phase separation and CFTR entry into platforms.** A7, M2 and HBE cells transduced with wt-CFTR adenovirus. Shortly before imaging, cells were treated with low concentration of SDS (100 µM) or Triton X-100 (25–80 µM) for 5–10 min. (A–C) Both detergents induced CFTR clustering in A7 cells that otherwise would lack clusters (SDS: *N*_exp_=6; *n*_cell_=281, TX100: *N*_exp_=3; *n*_cell_=112). (D–F) M2 cells form bright clusters at the PM following a treatment with either of the detergents (SDS: *N*_exp_=4; *n*_cell_=123, TX100: *N*_exp_=3; *n*_cell_=83). (G–I) Both detergents induce wt-CFTR-rich platforms in HBE cells resembling those after Thaps (SDS: *N*_exp_=5; *n*_cell_=187, TX100: *N*_exp_=4; *n*_cell_=138). White arrows highlight CFTR clustering; blue arrows highlight CFTR-rich platforms.

The ability of CFTR to form clusters and platforms independently of scaffold proteins suggests they may be driven by interactions with membrane lipids. To manipulate lipid order, we exposed cells to low (i.e. sub-micellar) concentrations of Triton X-100 and SDS, which partition preferentially into liquid-disordered membrane regions and promote the lateral separation of lipid domains (Staneva et al., 2005) in artificial systems (Carita et al., 2017) and live cells (Gok et al., 2020). Since CFTR clusters form spontaneously in HBE cells we used M2 and A7 cells (neural crest-derived melanoma cell lines without and with filamin A, respectively) to examine the effects of detergent on clustering. EGFP–CFTR was diffusely distributed in both M2 and A7 cells (Fig. 4A,D). Exposure to 100 µM SDS (Fig. 4B,E) or 80 µM Triton X-100 (Fig. 4C,F) for 10 min at 37°C induced CFTR clusters in these cells, consistent with the formation of lipid rafts. These results indicate that clusters are induced by detergents that promote lipid phase separation and may be sufficient to cause CFTR aggregation into raft-like microdomains. Exposing HBE cells with CFTR clusters to lower SDS or Triton X-100 (25 µM) concentrations yielded platforms within 3–5 min at 37°C (Fig. 4G–I), probably due to cell stress induced by the detergents (Borner et al., 1994).

### Overexpression allows some trafficking of misfolded CFTR to the PM

**Fig. 5.**
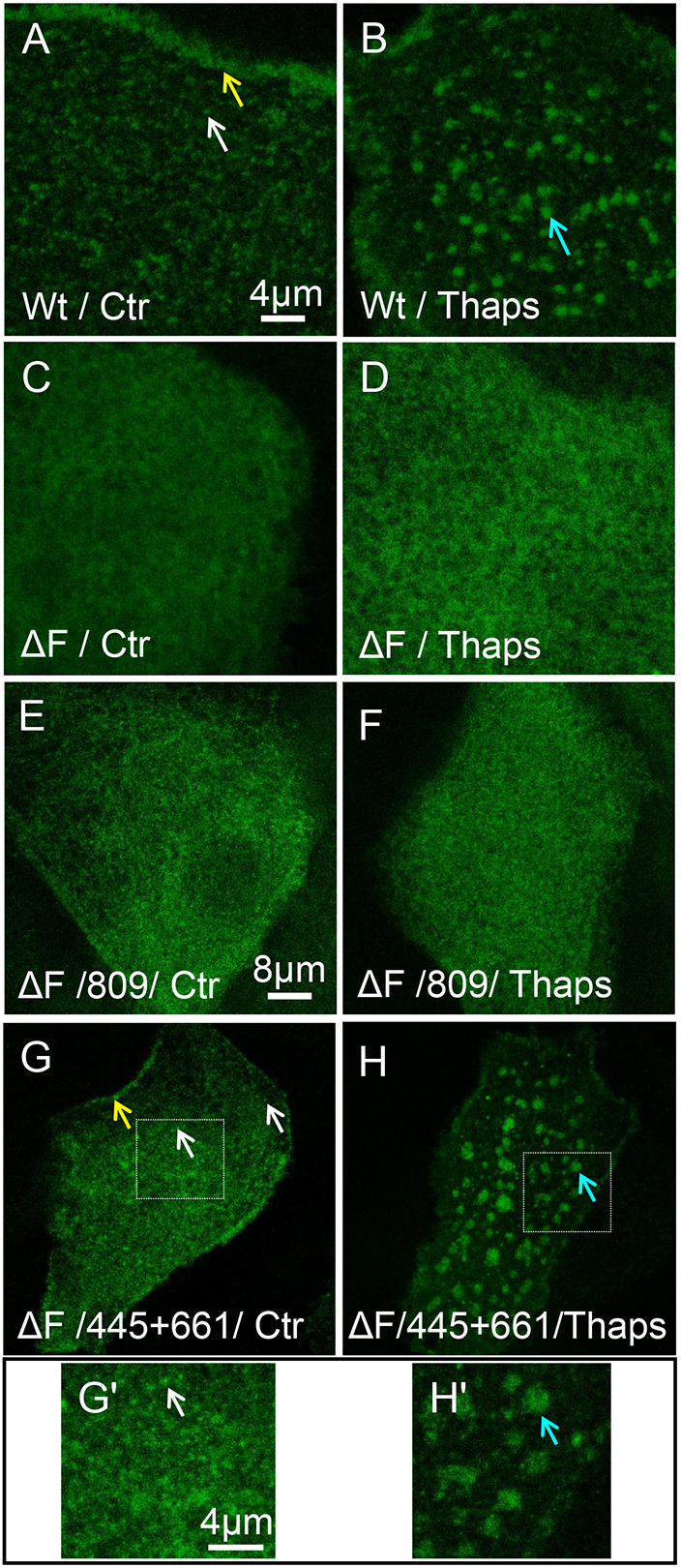
**Trikafta correctors restore F508del-CFTR clustering at the PM.** HBE cells transduced with adenoviruses containing wt- or F508del-CFTR. (A,B) PM distribution of wt-CFTR shows clusters (white arrow, *N*_exp_=10; *n*_cell_=380) and platforms after Thaps (blue arrow, *N*_exp_=8; *n*_cell_=156). CFTR is also enriched near cell–cell junctions (rim, yellow arrow). (C) F508del-CFTR lacks cluster and does not accumulate near junctions (*N*_exp_=14; *n*_cell_=315). (D) There is no recruitment of F508del-CFTR into platforms after Thaps treatment (*N*_exp_=5; *n*_cell_=147). (E,F) Lumacaftor (VX-809, 24 h) does not restore F508del-CFTR clustering or entry into platforms (*N*_exp_=6; *n*_cell_=213). (G,H) VX-445 plus VX-661 (24 h) restores F508del-CFTR membrane expression, clustering (white arrow) and rim formation (yellow arrow, *N*_exp_=19; *n*_cell_=842, see inset). Thaps treatment triggers formation of F508del-CFTR-enriched platforms (blue arrow, *N*_exp_=6; *n*_cell_=264, see inset), further evidence that corrected F508del-CFTR partitions inside lipid rafts. Magnified views of the indicated area are shown in G′,H′.

Adenoviral transduction produced relatively high F508del-CFTR expression and some surface expression, probably through saturation of ER quality control (ERQC) mechanisms (Sun and Brodsky, 2019). We compared the surface expression and clustering of wt-CFTR and F508del-CFTR constructs bearing a 3HA tag in the fourth extracellular loop. Immunostaining intact cells with extracellular anti-HA antibody yielded strong 3HA–wt-CFTR immunofluorescence (Fig. S5A) and a much weaker 3HA–F508del-CFTR signal at the cell surface (Fig. S5B). Individual 3HA–F508del-CFTR puncta were dim compared to 3HA–wt-CFTR clusters but could be detected by adjusting the fluorescence intensity levels (compare arrow in Fig. S5B,C). Autofluorescence did not contribute significantly to the signal measured at the cell surface even when high laser power was used (40%, Fig. S5D), although it was detectable intracellularly in non-transduced cells. The fluorescence intensity (*I*) of individual 3HA–F508del-CFTR puncta (*I*_ΔF_=0.44±0.03 arbitrary units, *n*_cell_=20 cells, *n*_cluster_=156 total clusters; mean±s.e.m.) was lower than that of wt-CFTR clusters (*I*_wt_=2.21±0.05; *n*_cell_=4, *n*_cluster_=118) suggesting they contained ∼5-fold fewer channels (Fig. S5F,G), and the number of 3HA–F508del-CFTR puncta was 4-fold lower compared to 3HA–wt-CFTR. Treating cells with VX-445 plus VX-661, correctors in the recent combination CF drug Trikafta, increased 3HA–F508del-CFTR immunofluorescence at the cell surface (compare Fig. S5E with Fig. S5C, see also Fig. 5G). Thus, overall surface expression of 3HA–F508del-CFTR was >20-fold lower than 3HA–wt-CFTR and was partially restored by VX-445 plus VX-661 (Fig. S5E–G).

### Clustering is defective in CF but restored by Trikafta correctors

**Fig. 6.**
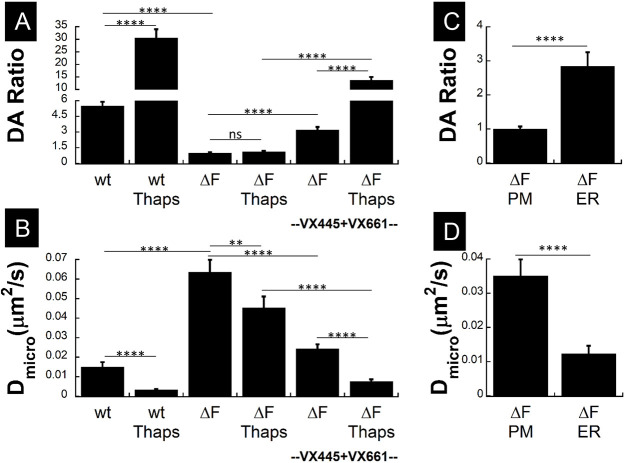
**Trikafta correctors restore F508del-CFTR clustering and confinement inside microdomains.** HBE cells transduced with wt- or F508del-CFTR adenoviruses. (A) Degree of aggregation (DA ratio) showing that wt-CFTR clusters are five times larger than F508del-CFTR. Thaps increases the wt-CFTR DA ratio due to incorporation in platforms whereas F508del-CFTR DA is unchanged. VX-445 plus VX-661 correction restores normal F508del-CFTR DA ratio and clustering. Thaps treatment increased the DA ratio, indicating entry of the mutant into platforms. (mean±s.e.m.; *N*_exp_=2; *n*_cell_, *n*_wt_=40, *n*_wt+Thaps_=37, *n*_ΔF_=108, *n*_ΔF+Thaps_=72, *n*_ΔF(VX)_=98, *n*_ΔF(VX)+Thaps_=95). ****P<0.0005; ns, not significant. (B) *D*_micro_ from k-space image correction spectroscopy analysis indicates confined mobility of CFTR inside microdomains and is higher for F508del-CFTR than wt-CFTR, indicating weaker confinement. Thaps reduces mobility and increases confinement due to the presence of wt-CFTR inside ceramide-rich platforms but does not affect F508del-CFTR, which is excluded from ceramide-rich platforms. VX-445 plus VX-661 correction partially restores F508del-CFTR mobility and confinement under Ctr and Thaps conditions (mean±s.e.m.; *N*_exp_=2; *n*_cell_, *n*_wt_=40, *n*_wt+Thaps_=37, *n*_ΔF_=91, *n*_ΔF+Thaps_=51, *n*_ΔF(VX)_=85, *n*_ΔF(VX)+Thaps_=80). ****P<0.0005; **P<0.025. (C,D). Differences in F508del-CFTR degree of aggregation (DA ratio) and confined mobility (*D*_micro_) in the PM and the ER indicate the presence of some weakly confined F508del-CFTR channels at the PM (mean±s.e.m.; *N*_exp_=2; *n*_cell_, *n*_ΔF(PM)_=39, *n*_ΔF(ER)_=30). ****P<0.0005. Unpaired one-tailed *t*-tests were used throughout the analysis, and each cell is an independent biological sample.

EGFP–wt-CFTR and EGFP–F508del-CFTR distributions were very different under both control and Thaps-stimulated conditions (compare Fig. 5A,B with C,D). Very few clusters and no platforms were observed on cells expressing F508del-CFTR (Fig. 5C,D; Fig. S5B). The first-generation corrector VX-809 (lumacaftor) did not restore F508del-CFTR clustering when cells were exposed to it alone or in combination with VX-770 (Fig. 5E,F; Fig. S6). Exposing cells to SDS or Triton X-100 also did not induce F508del-CFTR clustering or platforms, in contrast to wt-CFTR (compare Fig. S7A–C with Fig. 4G–I). However, the more potent corrector combination VX-445 plus VX-661 did restore F508del-CFTR clustering (Fig. 5G, white arrow) and incorporation into platforms (Fig. 5H, blue arrow), which were detected both qualitatively (Fig. 5G,H; for inset see G′,H′) and quantitatively (Fig. 6). Image correlation spectroscopy (ICS) and k-space ICS analyses confirmed that aggregation of F508del-CFTR (DA ratio; Fig. 6A) was reduced compared to wt-CFTR and the small spatial scale diffusive mobility was increased (D_micro_; Fig. 6B), consistent with loss of clustering and a change in the lipid environment. wt-CFTR clusters were also five times brighter than F508del-CFTR puncta, and Thaps increased the DA ratio of wt-CFTR but not F508del-CFTR (Kolin and Wiseman, 2007; Abu-Arish et al., 2015). These results confirm that CFTR must be in lipid rafts to be incorporated into platforms during stimulation (Fig. 6A). They also explain the more pronounced slowing effect of Thaps on the D_micro_ of wt-CFTR compared to the mutant (Fig. 6B). Since most F508del-CFTR is retained in the ER (Fig. S1), we examined whether fluorescence from the ER could explain the differences in DA ratio and D_micro_ obtained for F508del- and wt-CFTR. The DA ratio and D_micro_ of F508del-CFTR was compared from images obtained while focused on the cell surface or at the middle of cell (where most signal from the ER is located). The DA ratio for F508del-CFTR was lower while the D_micro_ was higher at the PM as compared to the ER (Fig. 6C,D) suggesting some non-clustered F508del-CFTR is in the PM. In summary, correctors increase F508del-CFTR clustering and reduce its microscopic scale lateral diffusion to near wt-CFTR levels under both control and Thaps-stimulated conditions (Fig. 6A,B).

**Fig. 7.**
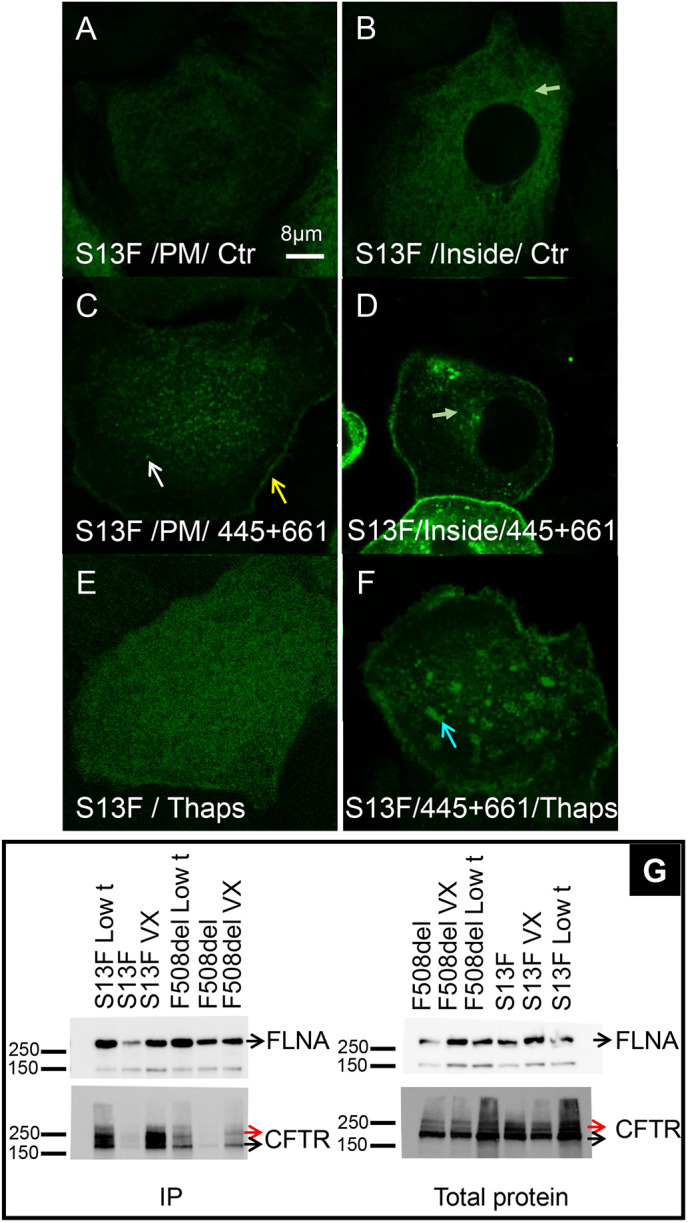
**Trikafta correctors restore S13F-CFTR folding.** HBE cells transduced with adenovirus containing S13F-CFTR. (A) Like F508del-CFTR, S13F-CFTR does not form clusters or a rim near junctions (*N*_exp_=11; *n*_cell_=341). (B) Mid-section through cell reveals that most S13F-CFTR is intracellular (green arrow). (C,D) VX-445 plus VX-661 correction reduces S13F-CFTR intracellular retention and restores membrane expression, clustering (white arrow) and rim formation (yellow arrow, *N*_exp_=16; *n*_cell_=582). The green arrow in D highlights the decrease in intracellular S13F-CFTR level after correction therapy. (E,F) S13F-CFTR fails to form platforms after Thaps treatment (*N*_exp_=3; *n*_cell_=90) (E), and Trikafta correctors ameliorate this defect (*N*_exp_=7; *n*_cell_=306) (F). The blue arrow in F highlights the restoration of S13F-CFTR incorporation in ceramide-rich platforms after correction therapy. (G) As shown by co-immunoprecipitation, VX-445 plus VX-661 (and low temperature, 29°C for 24 h) restores interaction of S13F-CFTR and F508del-CFTR with FLNA suggesting both mutants are misfolded and rescued by these correctors (*N*_exp_=3). 20 μg of protein were used for the lysate blot and 750 μg for the IP blot (2.7% of the IP).

The CF mutation S13F reduced CFTR surface expression, disrupted filamin A binding and prevented clustering (Fig. 7A). The intracellular distribution of S13F-CFTR resembled that of F508del-CFTR (Fig. 7B). Therefore we examined its response to Trikafta correctors. VX-445 plus VX-661 restored S13F-CFTR clustering and junctional localization (Fig. 7C, white and yellow arrow, respectively), and this was accompanied by a decrease in the intracellular pool (Fig. 7D). S13F-CFTR incorporation into platforms was restored by VX-445 plus VX-661 (compare Fig. 7E,F) as was FLNA binding (Fig. 7G). FLNA co-immunoprecipitated with both the immature and mature glycoforms of S13F-CFTR and F508del-CFTR after correction by VX-445 plus VX-661 (black and red arrows, see Fig. S7D for band density quantification); therefore disruption of the FLNA interaction apparently results from generalized misfolding of these mutants rather than disruption of a specific FLNA-binding site by mutagenesis (Fig. 7G). When CFTR constructs were expressed at comparable levels in HBE cells, the increase in short-circuit current (*I*_sc_) in response to forskolin plus genistein had the sequence wt-CFTR>S13F-CFTR>F508del-CFTR (Fig. S8A–F). The channel activity of both mutants was also partially rescued by VX-445 plus VX-661. These results indicate that correctors restore trafficking, clustering, platform formation and channel function of both F508del- and S13F-CFTR.

### CFTR modulates lipid rafts

**Fig. 8.**
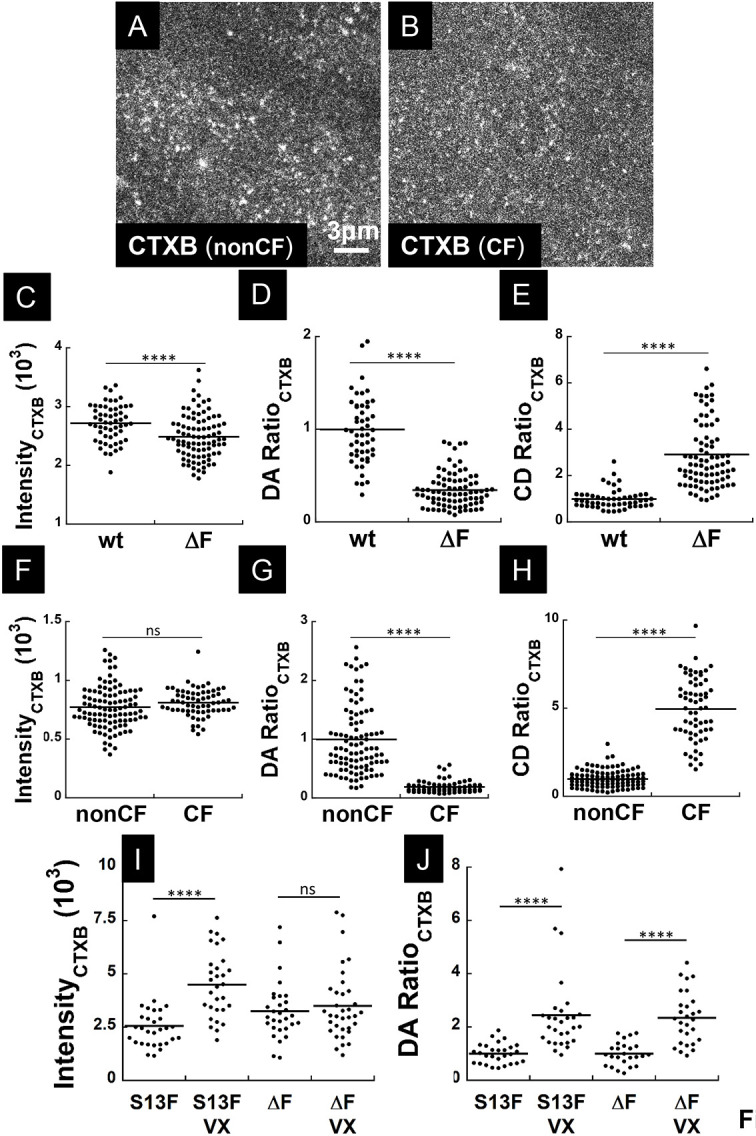
**Reciprocal effects of CFTR on membrane lipids.** Cells were exposed to CTXB conjugated to the fluorophore Alexa Fluor 594 (CTXB–594), which binds to the ganglioside GM1 and labels GM1-positive lipid rafts, at 0.5 μg/ml for 30 min before imaging. (A,B) CTXB–594 distribution at the PM of non-CF (A) and CF (B) cells showing GM1 clustering. (C–E) ICS analysis comparing distribution of GM1 clusters on HBE cells that overexpress wt- or F508del-CFTR (ΔF). (C) Total fluorescence due to CTXB–594 binding is similar on cells expressing wt- and F508del-CFTR. (D,E) GM1 aggregation (DA ratio) is 3-fold higher and cluster density (CD ratio) (# cluster per μm^2^) is 3-fold lower for wt-CFTR than F508del-CFTR, suggesting it promotes lipid raft formation. *I*_wt_(×10^3^)=2.73±0.04 arbitrary units (AU), *I*_ΔF_(×10^3^)=2.49±0.04 AU, DA_wt_ ratio=1.00±0.05, DA_ΔF_ ratio=0.35±0.02, CD_wt_ ratio=1.00±0.06, CD_ΔF_ ratio=2.9±0.2 (mean±s.e.m.; *N*_exp_=3, *n*_cell_, *n*_wt_=52, *n*_ΔF_=89). ****P<0.0005. (F–H) Similar results were obtained using untransduced cells expressing endogenous wt- (nonCF) or F508del-CFTR (CF). Overall CTXB fluorescence was similar (F); however, CTXB clusters were 5-fold larger (H) and their number per unit area was ∼5-fold lower on non-CF cells (G). *I*_nonCF_(×10^3^)=0.77±0.02 AU, *I*_CF_(×10^3^)=0.81±0.01 AU, DA_nonCF_ ratio=1.00±0.07, DA_CF_ ratio=0.19±0.01, CD_nonCF_ ratio=1.00±0.05, CD_CF_ ratio=5.0±0.2 (mean±s.e.m.; *N*_exp_=3; *n*_cell_, *n*_nonCF_=100, *n*_CF_=62). ****P<0.0005; ns, not significant. (I,J) Trikafta correctors (VX) increase aggregation state of GM1-positive rafts (CTXB clusters) by ∼2.5-fold when HBE cells overexpress F508del-CFTR or S13F-CFTR [mean±s.e.m. *N*_exp_=2, *n*_cell_: *n*_S13F_=30, *n*_S13F(VX)_=30, *n*_ΔF_=30, *n*_ΔF(VX)_=25]. ****P<0.0005; ns, not significant. Each point on the histogram represents a cell, and each cell is an independent biological sample. Unpaired one-tailed *t*-tests were used throughout the analysis.

The PM contains most of the total wt-CFTR protein in HBE cells and most cellular sphingolipid and cholesterol, therefore we examined whether CFTR deficiency at the cell surface affects the formation of membrane microdomains. We used cholera toxin subunit B conjugated to Alexa Fluor 594 (CTXB–594) to visualize rafts containing the ganglioside GM1 (Lingwood et al., 2008; Hullin-Matsuda and Kobayashi, 2007). Although total CTXB–594 binding was similar on cells expressing wt-CFTR or F508del-CFTR (Fig. 8C), patches of CTXB–594 fluorescence were brighter and less numerous on wt-CFTR cells, with higher measured degree of aggregation (DA ratio, #CTXB per cluster; Fig. 8D) and lower cluster density ratio (CD ratio, #clusters per µm^2^; Fig. 8E) for CTXB–594. Similar results were obtained when CF and non-CF HBE cells expressing only endogenous CFTR were compared (Fig. 8F); that is, non-CF cells had larger CTXB–594 aggregates (Fig. 8G,H). VX-445 plus VX-661 correction of HBE cells expressing F508del- or S13F-CFTR increased the aggregation state of CTXB–594 by ∼2.5-fold (Fig. 8J) indicating that pharmacological rescue of mutated CFTR promotes the fusion of GM1-positive rafts. These results suggest that the formation of membrane microdomains is inhibited by reduced CFTR expression at the cell surface.

## DISCUSSION

In this study, we examined CFTR incorporation into lipid rafts and ceramide-rich platforms and the impact of disease-causing mutations (Abu-Arish et al., 2019, 2015). CFTR clustering did not require interaction with known scaffold proteins but instead appeared lipid driven, since it was induced by low concentrations of detergents that enhance raft formation by dissolving preferentially in disordered membrane regions. Clustering and platform incorporation were abrogated by the severe CF mutations F508del and S13F and restored by Trikafta correctors. The presence of CFTR in the PM increased the size and number of lipid rafts containing the ganglioside GM1. Clustering enables CFTR to enter long-lived ceramide-rich platforms when cells are stimulated (Abu-Arish et al., 2019), which promotes CFTR accumulation and may also facilitate functional interactions with other proteins (Kunzelmann, 2001). Since physiological agonists, such as VIP, signal through multiple pathways, for example, by elevating both cAMP and Ca^2+^, clustering and platform-dependent stabilization are likely to enhance the efficacy of CF corrector drugs (Langer, 2012; Abu-Arish et al., 2019).

### Lipid interactions drive clustering independently of scaffold proteins

The sensitivity of CFTR clustering to treatments that deplete or supplement cholesterol provided indirect evidence that clusters are situated in lipid rafts (Abu-Arish et al., 2015), and this is supported by the present results showing a close association of CFTR clusters with BODIPY analogs of both cholesterol and C12-sphingomyelin. Localization in rafts is also consistent with the early demonstration of CFTR in a detergent-resistant membrane fraction prepared from airway epithelial cell lines (Dudez et al., 2008; Wang et al., 2008). Since EGFP–F508del-CFTR did not form clusters in cells that also expressed endogenous wt-CFTR (data not shown) while G551D-CFTR clustered normally in CF cells expressing endogenous F508del-CFTR, the inability of F508del-CFTR to cluster was not a secondary consequence of the CF cellular phenotype.

To explore the mechanism of clustering, we tested several well-established CFTR-protein interactions. We began with PDZ-domain proteins because NHERF1 and NHERF2 can tether CFTR to the cytoskeleton through the actin-binding protein ezrin (Bates et al., 2006; Haggie et al., 2004, 2006; Valentine et al., 2012; Short et al., 1998). NHERF1 causes transient confinements and occasional long-lived immobilizations of CFTR that can be detected by single-particle tracking (Bates et al., 2006; Haggie et al., 2006), and also reduces CFTR lateral mobility as measured by fluorescence recovery after photobleaching (Bates et al., 2006; Haggie et al., 2004) and ICS (Bates et al., 2006). Moreover NHERF1 multimerizes and has a cholesterol-binding motif that could potentially target CFTR to lipid rafts (Sheng et al., 2012). However, deleting the PDZ-binding motif from the C-terminus of CFTR did not affect clustering, although co-immunoprecipitation with NHERF1 was abolished (Bates et al., 2006). FLNA is another scaffold protein that forms homodimers and mediates clustering of other membrane proteins. Although FLNA has elongated Ig-repeats that bind multiple CFTR molecules and stabilize them at the PM (Ithychanda et al., 2009; Smith et al., 2010; Playford et al., 2010; Thelin et al., 2007), normal CFTR clusters formed in FLNA-deficient HBE cells. Actin has been reported to bind CFTR (Chasan et al., 2002); however, disrupting the actin cytoskeleton using latrunculin B and cytochalasin D also did not prevent cluster formation. Taken together, these results indicate interactions with scaffold proteins play little role in clustering. We considered the possibility that lipidation of CFTR helps target it to rafts (Melkonian et al., 1999); however, mutating the major palmitoylation site on CFTR at C1395 or inhibiting palmitoylation generally with the inhibitor 2BP did not affect its clustering, prompting us to investigate CFTR interactions with membrane lipids.

Nanoscale lipid rafts are dynamic, liquid-ordered domains that are enriched in sphingolipids and raftophilic proteins (Simons and Ikonen, 1997; Sezgin et al., 2017). Cholesterol is associated with sphingolipids, although the long-held view that cholesterol is enriched in rafts has been challenged (Frisz et al., 2013). We manipulated membrane order using low concentrations of detergent (25–100 µM SDS or Triton X-100) as this promotes lipid raft formation by preferentially dissolving in the liquid-disordered domain and increasing lipid phase separation (Staneva et al., 2005). Detergents induced CFTR cluster formation on M2 and A7 cells that normally lack them. Together with our previous results manipulating cholesterol levels, these findings indicate that CFTR clustering may be driven by CFTR–lipid rather than CFTR–protein interactions.

Loading HBE cells with BODIPY-cholesterol revealed that CFTR partitions into microdomains that accumulate this cholesterol analog. Although cholesterol levels are thought to decline in microdomains when sphingomyelin is cleaved to ceramide (Yu et al., 2005), elevated levels of both BODIPY-C12-sphingomyelin and BODIPY-cholesterol were observed in ceramide-rich platforms. Another interesting observation was the recovery of CFTR platforms in the PM of A7 and M2 cells after cholesterol supplementation (data not shown), suggesting cholesterol deficiency may contribute to the inability of CFTR to form clusters and platforms in these cell lines. A similar deficiency might also explain why CFTR does not cluster efficiently in cell lines such as baby hamster kidney fibroblasts (BHK) and human embryonic kidney (HEK) cells.

### F508del and S13F prevent CFTR clustering and entry into ceramide-rich platforms

Most F508del CFTR was retained intracellularly as expected; however, adenoviral expression varied over a wide range, enabling cells with comparable levels of wt and mutant protein to be studied by selecting cells with relatively low wt-CFTR and high F508del-CFTR expression. F508del-CFTR and S13F did not form clusters when expressed in CF or non-CF HBE cells, although immunostaining with an extracellular 3HA tag (i.e. 3HA–F508del-CFTR) revealed F508del-CFTR at the PM. Differences in the degree of aggregation and lateral mobility of F508del-CFTR in the PM versus the ER further support that F508del-CFTR is present on the cell surface following adenoviral transduction. Thus, while mis-trafficking and low surface expression of the mutants contributes to their inability to form clusters and platforms, abnormal folding apparently also interferes with lipid interactions at the cell surface, perhaps due to mismatch between hydrophobic transmembrane segments of CFTR and raft lipids (Killian, 1998) or impaired sphingolipid or cholesterol binding (Nyholm, 2015). CFTR transmembrane segments do not possess a signature motif for sphingolipid binding (VXXTLXXIY) (Contreras et al., 2012; Bjorkholm et al., 2014). They have multiple CRAC and CARC consensus sequences for cholesterol binding (Lorent and Levental, 2015); however, whether cholesterol binding occurs at these sites and is altered in F508del-CFTR remains to be determined.

### Effect of CF therapeutics on CFTR clustering and incorporation into platforms

The success of these therapeutics in correcting CFTR folding, trafficking, membrane localization and function provides proof-of-principle for the development of therapeutics for other protein trafficking diseases; therefore it is important to understand their mechanisms of action, including the roles of protein–protein and protein–lipid interactions. Treatment with VX-809 increased the fraction of cells expressing detectable EGFP–F508del-CFTR fluorescence but did not restore EGFP–F508del-CFTR clustering at the PM. By contrast, more efficacious correction by VX-445 plus VX-661 restored clustering and lateral mobility. Similar results were obtained with S13F-CFTR, which is a mutation in the N-terminal lasso motif of CFTR. We confirmed that mutating the small and polar serine residue to phenylalanine abolishes its ability to co-immunoprecipitate FLNA and reduces S13F-CFTR expression at the PM (Thelin et al., 2007). F508del and S13F caused surprisingly similar inhibition of clustering, which was reversed by VX-445 plus VX-661, suggesting both mutations have similar defects in protein conformation. This is further suggested by the ability of VX-445 plus VX-661 to rescue FLNA coprecipitation with both S13F- and F508del-CFTR. In summary, both mutants were retained predominantly in the ER, did not form clusters despite the presence of some protein in the PM, failed to co-precipitate with FLNA and were corrected similarly by VX-445 plus VX-661.

### GM1-positive membrane microdomains are larger when wt-CFTR is expressed

Fluorescent cholera toxin B subunit (CTXB–594) bound to CF and non-CF cells with similar efficiency suggesting that they have a comparable overall abundance of GM1. However, image correlation spectroscopy revealed 5-fold lower GM1 aggregation on CF cells, indicating that endogenous wt-CFTR promotes the formation of larger rafts. GM1-positive rafts were also enlarged in HBE cells overexpressing wt-CFTR compared to cells expressing F508del-CFTR, and Trikafta correctors increased the size of GM1-positive rafts by 2.5-fold. The apparent lack of clusters and platforms may be explained by smaller rafts. The mechanism by which CFTR modulates raft size remains to be determined; however, cholesterol (White et al., 2007) and the ω-3 polyunsaturated fatty acid DHA (Freedman et al., 1999) may be relevant, as they influence raft stability in model and biological membranes and their levels are abnormal in CF (Levental et al., 2016).

In summary, CFTR clustering occurs through a mechanism that depends mainly on interactions with lipids rather than other proteins. Clustering enables CFTR incorporation into ceramide platforms but is prevented by some CF-causing mutations. Potent correctors of CFTR misfolding such as those in Trikafta restore CFTR clustering and dynamics at the cell membrane, a previously unrecognized mechanism of action that may be relevant to other protein folding diseases.

## MATERIALS AND METHODS

### Cell culture

Primary human bronchial epithelial cells (HBEs) were obtained from the Cystic Fibrosis Canada Primary Airway Cell BioBank at McGill University (https://mcgill.ca/cftrc/platforms/primary-airway-cell-biobank-pacb). Human subjects were not used in this study, only cells isolated from tissues discarded after lung transplantation were used. CF lung tissues were from the Respiratory tissue Biobank at the Centre hospitalier de l'Université de Montréal Research Centre (CRCHUM) and were obtained with informed consent following protocols approved by the Institutional Review Boards at the CRCHUM and McGill University (#A08-M70-14B). Non-CF lungs were obtained from the National Development and Research Institutes, Inc. (NDRI, New York, NY) and the International Institute for the Advancement of Medicine (IIAM, Edison, NJ). Isolated cells were tested for mycoplasma contamination. For live-cell imaging, HBE cells were seeded at first passage in polycarbonate FluoroDishes (23.5 mm diameter optical glass bottom; World Precision Instruments, Inc., Sarasota, FL) pre-coated with collagen (PureCol, Advanced BioMatrix, San Diego, CA) and transduced with adenoviruses at a multiplicity of infection (MOI) of 30 to express wt-CFTR and various CFTR mutants fused to EGFP as described previously (Abu-Arish et al., 2015; Vais et al., 2004). A7 and M2 cells, a kind gift from Dr Reza Sharif-Naeini, were maintained in Eagle's minimum essential medium (Wisent) supplemented with 10% FBS (Wisent), 1% penicillin-streptomycin (Wisent) and 1% L-glutamine (Wisent).

### Mutagenesis and adenoviral vector construction

EGFP–wt-CFTR in pShuttle2 was provided by the Penn Vector Core, Gene Therapy Program, Perelman Sch. Med., Univ. Pennsylvania, Philadelphia, PA. EGFP–ΔPDZ-CFTR, EGFP–S13F-CFTR, EGFP–C1395A-CFTR and EGFP–G551D-CFTR cDNAs were generated by Mutagenex (Columbus, OH). EGFP was replaced with mCherry by GenScript (Piscataway, NJ). Three hemagglutinin epitopes (YPYDVPDYAAE) were inserted in the fourth extracellular loop of wt-CFTR and F508del-CFTR after amino acid 901, bracketed by GC and CG linkers. Mutants were confirmed by Sanger sequencing then subcloned into pAdeno-X vector (Clontech, Mountain View, CA) using the In-Fusion reaction following the manufacturer's instructions, and were confirmed by Sanger sequencing. pAdeno-X DNA was purified and linearized, and transfected into Adeno-X 293 cells stably expressing Ad5E1 genes using Lipofectamine 2000. Floating cells were collected for lysis and reinfection. Repeating this cycle five times yielded 2–5 ml stock virus with 3–15 MOI/µl (MOI, multiplicity of infection).

### Filamin A knockdown

Gene silencing was performed by reverse transfection of HBE cells (CF and non-CF) with Dicer siRNA (DsiRNA, Integrated DNA Technologies; Ramachandran et al., 2013). Dicer siRNA was diluted in OptiMEM (Gibco), combined with Lipofectamine RNAiMAX (ThermoFisher) and added to the wells of collagen IV-coated FluoroDishes. HBE cells were resuspended in Dulbecco's modified Eagle's medium (DMEM/F12) supplemented with 10% heat-inactivated fetal bovine serum (FBS), seeded on top of the transfection mixture, and incubated overnight at 37°C. The following day, the medium was replaced with DMEM/F12 plus 10% heat-inactivated FBS for 6 h before cells were infected with adenovirus according to the previously detailed infection procedure. The DsiRNAs used were as follows: Scrambled DsiRNA, sense strand, 5′-CUUCCUCUCUUUCUCUCCCUUGUGA-3′, antisense strand, 5′-AGGAAGGAGAGAAAGAGAGGGAACACU-3′; filamin A DsiRNA, sense strand, 5′-GUUUACCUGAUUGACGUCAAGUUCA-3′, antisense strand, 5′-UGAACUUGACGUCAAUCAGGUAAACGC-3′.

RNA was extracted from HBE cells using the Illustra RNA Spin Mini kit (GE Healthcare) according to the manufacturer's instructions. 250 ng RNA was reverse transcribed with 5× All-In-One RT MasterMix (ABM) in a reaction volume of 20 µl by incubation at 25°C for 10 min, 42°C for 15 min and 85°C for 5 min. qPCR was performed by adding 0.5 µl cDNA, 10 μl of TaqMan^®^ Fast Advanced Mastermix, 1 μl of TaqMan Gene Expression Assay primers in a reaction volume of 20 μl to the wells of a MicroAmp^®^ EnduraPlate™ Optical 96-Well Fast Reaction Plate. The reaction was carried out using a QuantStudio 7 Flex Real-Time PCR system (Applied Biosystems, Foster City, CA) using the following protocol: 20 s at 95°C and 40 cycles at 95°C (1 s) and 60°C (20 s). ΔΔCT analysis was performed using the manufacturer's software package.

### Cell treatments

Cells were incubated with adenovirus particles (MOI=30–50) for 2 days then removed by rinsing with OptiMEM. Imaging was performed 2–3 days later. Clusters were imaged when HBE cells were bathed in control medium (OptiMEM) and after 10–20 min exposure to 2 μM thapsigargin (Thaps, Sigma) to elevate intracellular Ca^2+^ and induce platforms. Cells were transduced with adenoviral wt-CFTR or F508del-CFTR bearing a 3HA-tag in the fourth extracellular loop for extracellular immunolabeling. HBE cells expressing mCherry–wt-CFTR were loaded with 1–2 µM BODIPY-cholesterol (Cayman Chem., Ann Arbor, MI) using methyl-β-cyclodextrin (MβCD; Sigma, Oakville, ON) at 1:10 cholesterol:MβCD molar concentration as a carrier for 2 min before imaging. Alternatively, cells were loaded with 0.5 µg/ml BODIPY-C12-sphingomyelin (Invitrogen, Carlsbad, CA) for 5 min before adding thapsigargin to induce ceramide platform formation. Microdomains containing the ganglioside GM1 were visualized in 80% confluent HBE cells expressing EGFP–wt-CFTR by incubating with 0.5 μg/ml CTXB–594 (Alexa Fluor 594-tagged cholera toxin-B subunit, Invitrogen) for 20–30 min immediately before imaging. In some experiments, cells expressing CFTR mutants were treated with 1 μM lumacaftor (VX-809, Selleckchem, Houston, TX) in the presence or absence of the potentiator ivacaftor (VX-770, 100 nM, MedChemExpress, Monmouth Junction, NJ) or with a combination of elexacaftor (VX-445, 3 μM, MedChemExpress) and tezacaftor (VX-661, 3 μM, MedChemExpress) for 24 h prior to imaging. To disrupt the cytoskeleton, cells were exposed to 0.15 or 0.3 μM latrunculin B (LatB, Abcam) or 0.5 μM cytochalasin D (CytoD, Abcam) for 10–15 min. To label the actin cytoskeleton, cells were stained with Alexa Fluor™ 594 phalloidin (Invitrogen) at 1 unit per sample for 5 min before washing and fixation. To induce liquid-ordered domains, cells were treated with SDS (100 µM, BioShop) or Triton X-100 (25–80 µM, Sigma) for 5–10 min before imaging. To inhibit CFTR palmitoylation, cells expressing 3HA–wt-CFTR were treated with the general palmitoylation inhibitor 2-bromo-palmitoyl (2BP, Millipore Sigma) at 5 or 10 µM for 2, 5, or 24 h.

### Live-cell imaging

Live cells were imaged at 37°C in a humidified incubator in 5% CO_2_/95% air (Live Cell Instrument, Seoul, South Korea) on the stage of an LSM780 confocal microscope as described previously (Abu-Arish et al., 2015). For image correlation spectroscopy (ICS) and k-space ICS (kICS) analyses, image time-series comprising 800 regions of interest (ROI, 256×256 pixels) were collected from a flat area of the PM in contact with the coverslip using a Plan-Apochromat 63× (NA=1.40) oil immersion objective with a confocal pinhole of 1 Airy unit, digital gain=900, 1% laser power, 6.5 Hz frame rate, pixel diameter=0.064 μm, and pixel dwell time of 1 μs as described previously in detail (Abu-Arish et al., 2015, 2019). For visualization, images of 512×512 or 1024×1024 pixels were collected at 5% laser power and a frame rate of 0.2 Hz (12 μs dwell time). EGFP-tagged CFTR mutants, HA-tagged wt- and F508del-CFTR, BODIPY-Cholesterol and BODIPY-C12-Sphingomyelin were imaged using the 488 nm Argon laser (25 mW). CTXB–594, Alexa Fluor^TM^ 594 phalloidin, FLNA immunostaining and mCherry–wt-CFTR were imaged using the 561 nm laser line (15 mW).

### Immunostaining

To immunolabel CFTR at the PM, CF HBE cells were transduced with wt-CFTR or F508del-CFTR containing a 3HA-tag in the fourth extracellular loop of CFTR. Confluent cells were gently washed with PBS and incubated with the mouse anti-HA.11 epitope tag antibody (1:400, Biolegend, cat. #901514) at 4°C for 45 min, then rinsed with PBS and fixed in 4% methanol-free formaldehyde (Thermo Fisher Scientific) for 15 min at room temperature. The fixed cells were exposed to goat anti-mouse-IgG conjugated to Alexa Fluor 488 secondary antibody (Invitrogen; 1:1000 dilution) for 1 h, washed with PBS and mounted in ProLong Diamond Antifade Mountant (Invitrogen) for imaging. The antibody was validated in cells that do not express any 3HA-tagged protein. For FLNA immunolabeling, and following FLNA knockdown, HBE cells transduced with wt-CFTR were gently washed with PBS, fixed, permeabilized (0.5% Triton X-100), blocked for 1 h (2% BSA) and incubated with the rabbit anti-FLNA antibody (1:100, Invitrogen, cat. #PA5-86143) for 2 h. Cells were then exposed to goat anti-rabbit IgG conjugated to Alexa Fluor 594 secondary antibody (Invitrogen; 1:1000 dilution) for 1 h and washed and mounted in PBS for imaging. The low fluorescence detected in cells due to the knockdown of FLNA (compared to the scrambled control) validated the antibody.

### Co-immunoprecipitation

CF HBE cells were infected with adenoviruses (wt-CFTR, F508del-CFTR or S13F-CFTR, 50 MOI), cultured to ∼90% confluency, and exposed to the Trikafta correctors VX-445 (3 μM, MedChemExpress) and VX-661 (3 μM, MedChemExpress), or maintained at low temperature (29°C) for 24 h (in OptiMEM). Cells were rinsed twice with ice cold PBS and lysed [150 mM NaCl, 1 mM EDTA, 50 mM Tris-HCl pH 7.4, 0.5% Triton X-100 and one tablet of protease inhibitor cocktail (Roche)]. Lysates were centrifuged (16,200 ***g*** for 20 min) at 4°C and total protein content was measured using Pierce™ BCA protein assay (Thermo Scientific). Aliquots containing 700–1000 μg protein were precleared with 20 μl Protein G Sepharose™ 4 Fast Flow beads for 30 min at 4°C then incubated with the rabbit anti-FLNA antibody (1:500 dilution, Invitrogen) for 30 min at 4°C. Immunocomplexes were precipitated using Protein G Sepharose beads, washed five times with lysis buffer, eluted using 2× Laemmli buffer, and analyzed by immunoblotting. The CFTR monoclonal antibody 596 (1:1000, CFFT clone#A4) was used, and was validated in Baby Hamster Kidney (BHK) cells by immunoblotting lysates of the cells with or without transfection with wt-CFTR.

### Short-circuit current

The short-circuit current (*I*_SC_) was measured in Ussing chambers using HBE CF cells transduced with wt-, F508del-, PDZdel- or the S13F-CFTR and seeded on collagen IV-coated polyester membrane inserts (Corning Costar 0.4 µM pore dia., 6.5 mm dia.). Cells were submerged for 4 days for recovery, then the medium was aspirated and cells were kept at the air–liquid interface (ALI) for another 7 days before functional assays. *I*_sc_ was measured as described previously (Matthes et al., 2016). Briefly, inserts were mounted in modified Ussing chambers (Physiologic Instruments Inc., San Diego, CA) and maintained at 37°C. The basolateral solution contained (in mM): 115 NaCl, 25 NaHCO_3_, 1.2 MgCl_2_, 1.2 CaCl_2_, 2.4 KH_2_PO_4_, 1.24 K_2_HPO_4_ and 10 D-glucose. The apical saline solution contained (in mM): 1.2 NaCl, 115 Na-gluconate, 25 NaHCO_3_, 1.2 MgCl_2_, 4 CaCl_2_, 2.4 KH_2_PO_4_, 1.24 K_2_HPO_4_ and 10 D-glucose. Cultures were maintained under voltage clamp except during 2 s voltage steps to ±1 mV at 100 s intervals to monitor resistance (Rt). Output from the voltage clamp amplifier (VCC200, Physiologic Instruments, Inc.) was digitized (Powerlab 8/30, AD Instruments) and analyzed using Chart5 software. The change in *I*_sc_ (Δ*I*_sc_) was measured after sequential apical additions of 100 µM amiloride, 10 µM forskolin (FSK), and genestein (Gen, 50 µM) or VX-770 (100 nM). Apical CFTR_inh_-172 (10 µM) and ATP (100 µM) were added sequentially on the apical side to confirm the currents CFTR dependence and viability, respectively.

### Image correlation spectroscopy analyses

Spatial image correlation spectroscopy (ICS) (Wiseman and Petersen, 1999) and k-space ICS (kICS) (Kolin et al., 2006) analyses are described in detail elsewhere (Abu-Arish et al., 2015). ICS was used to quantify the cluster density (CD; average number of independent fluorescent entities per unit area, #clusters/µm^2^) and the degree of aggregation (DA; proportional to the number of fluorescent labels per cluster), which are calculated as:
(1)

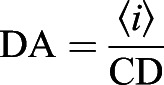

(2)

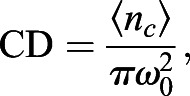
where 〈*i*〉 is the spatial average pixel intensity of the image region of interest (ROI), 〈*n*_*c*_〉 is the average number of clusters (fluorescent entities) per beam focal spot, and *ω*_0_ is the *e*^−2^ beam radius at focus. The 〈*n*_*c*_〉 is the reciprocal of the zero spatial lags amplitude of the spatial correlation function of the image, which is obtained from a nonlinear least squares fit of a 2D Gaussian function to the correlation function.

The kICS analysis was used to measure the microscopic (small spatial scales) diffusion coefficient *D*_micro_, which describes CFTR confined mobility inside domains. Briefly, the normalized k-space time correlation function is defined in the spatial frequency k (reciprocal space) as follows (Abu-Arish et al., 2015):
(3)

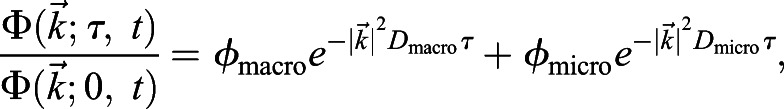
which is the sum of the diffusive transport contributions of the mobile fluorescent particle or cluster populations on short (micro) and long (macro) spatial scale (the macro- and micro-spatial scale dynamic populations). *φ*_*macro*_ and *φ*_*micro*_ are the amplitudes of the micro and macro correlation function components, and are proportional to the micro and macro population fractions, while D_macro_ and D_micro_ are their corresponding effective diffusion coefficients.

The normalized correlation function in Eqn. 3 is fit to a sum of two Gaussians at each temporal lag *τ*. The fit parameters are D_macro_*τ*, D_micro_*τ*, *φ*_macro_, and *φ*_micro_ as a function of *τ*. Mean-square displacement (MSD) plots for the two populations are then constructed from the fit-extracted *D_i_τ* versus *τ* for micro- and macro-scale transport.

In this study, the slope of the first three temporal lags of the micro MSD versus *τ* plot is calculated as *D*_micro_, which is the diffusion coefficient of particles confined inside the microdomains on cells. Fitted data were only rejected when the fit value returned a NaN. Software codes are available upon request from A.A.-A. or E.P.

### Statistics

Results are presented as the mean±s.e.m. for *n* cells. Parameters measured during treatments were compared to corresponding values in control cells using a unpaired one-tailed Student's *t*-test. DA Ratio and *D*_micro_ were measured using ICS and kICS, respectively. Since ICS and kICS measure the distribution (DA) and mobility (D_micro_) of single molecules at the spatial level of the cluster (≤ beam focal spot), and since each cellular measurement is the average of thousands of trajectories of molecules, each cell is considered an independent biological repeat. The results from 20 cells (biological repeats) were sufficient to show a significant difference in measured parameters under different treatment conditions. However, a larger number of analyses were performed with multiple preparations to ensure a normal data distribution. 3HA-tagged CFTR cluster brightness was measured using ImageJ. For *n* and *P-*values, please check figure legends (*N*_exp_, number of experiments; *n*_cell_, number of cells; *N*_patient,_ number of samples from patients).

## Supplementary Material

Supplementary information

Reviewer comments
