# Supplementary material for: Lipid-driven CFTR clustering is impaired in cystic fibrosis and restored by corrector drugs
Source: J Cell Sci. 2022 Mar 7;135(5):jcs259002. doi: 10.1242/jcs.259002 (PMC8976878; doi:10.1242/jcs.259002)
Supplement: Supplementary information [file joces-135-259002-s1.pdf]

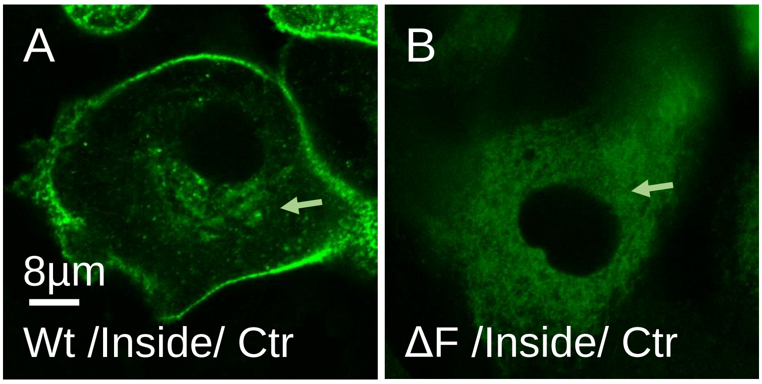

Fig. S1. Intracellular distribution of CFTR. Most EGFP-wt-CFTR fluorescence was observed at the plasma membrane in contrast to EGFP-F508del-CFTR, which was mostly intracellular.

Journal of Cell Science • Supplementary information

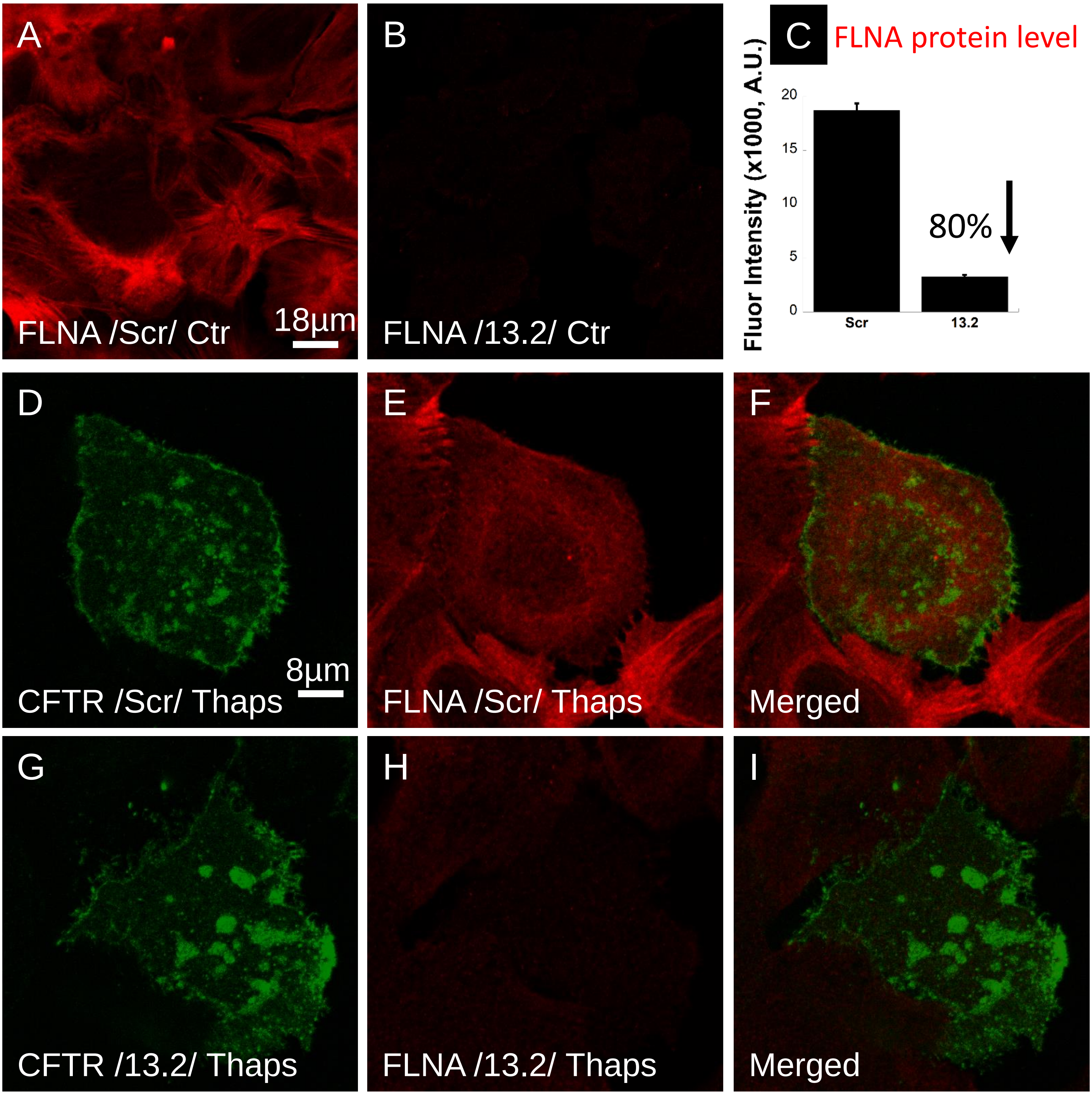

Fig. S2. Silencing FLNA does not affect CFTR entry into platforms. HBE cells transduced with EGFP-wt-CFTR adenovirus were immunolabeled against FLNA. (A) Endogenous FLNA distribution near the plasma membrane. (B) Successful FLNA knockdown (>90% reduction in the RNA level) using DsiRNA (13.2). (C) Based on the fluorescence intensity of the immunolabeling against FLNA, 80% reduction in FLNA protein expression was measured post knockdown (# of regions of interest (ROI) analyzed, n_{scr} = 20 & $n_{13.2}$ = 50, each ROI contained 2 – 3 cells). (D – F) CFTR entry into ceramide-rich platforms following thaps treatment. (G – I) In the absence of FLNA expression, Thaps-induced CFTR entry into platforms was not affected. Taken together, FLNA is not involved in CFTR platform formation.

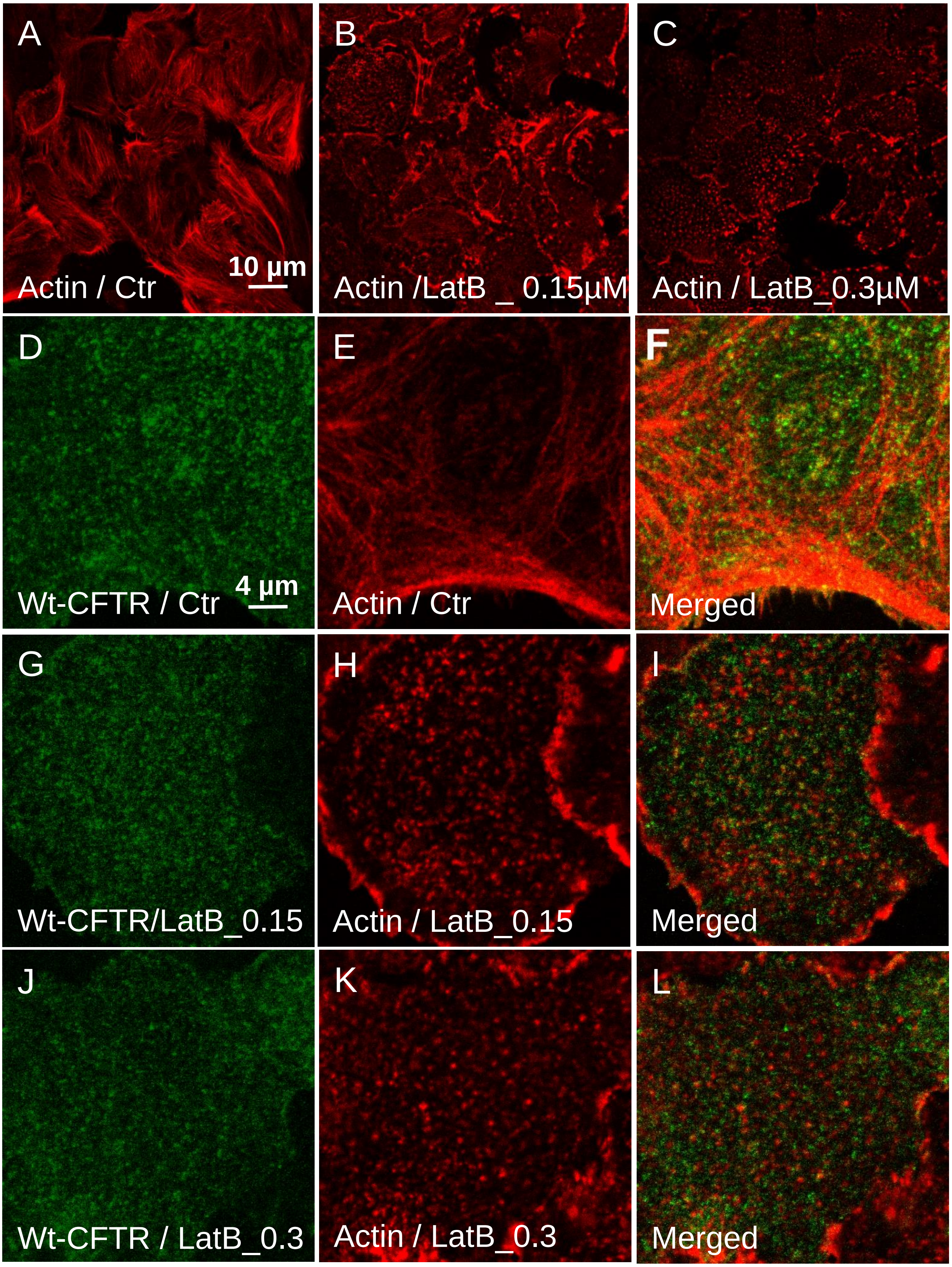

Fig. S3. Actin-independent CFTR clustering. HBE cells transduced with EGFP-wt-CFTR adenovirus were stained with phalloidin-594 (Actin). (A) Intact actin cytoskeleton distribution near the plasma membrane. (B & C) Concentration-dependent disruption of the actin cytoskeleton using LatB (0.15 & 0.3 µM). (D – L) CFTR clustering is independent of the actin cytoskeleton organization.

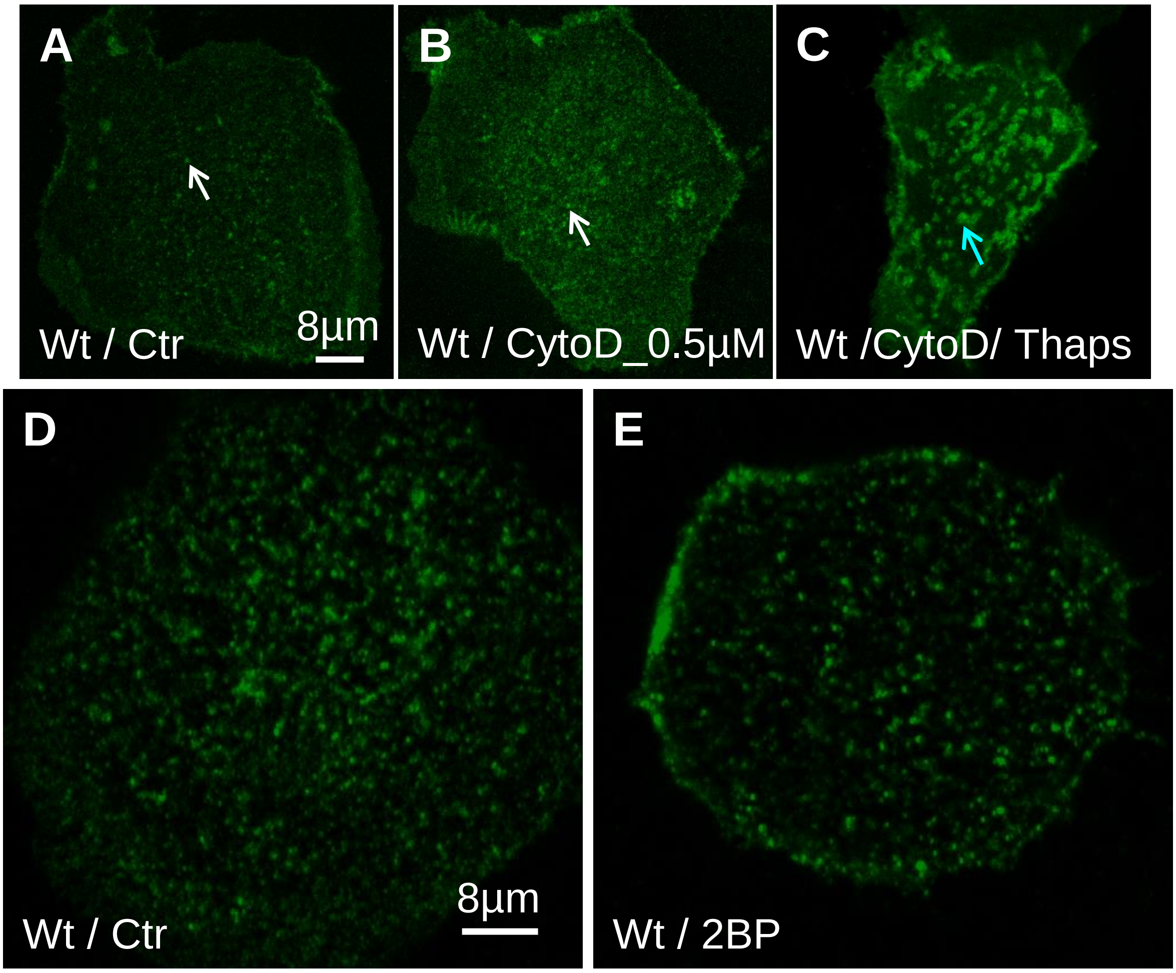

Fig. S4. Intact actin cytoskeleton or palmitoylation are not required for wt-CFTR clustering or entry into platforms. HBE cells were transduced with an adenovirus containing wt-CFTR. (A & B) Similar distribution of EGFP-wt-CFTR under Ctr conditions (N_{exp} = 12 & N_{cell} = 260) and after the actin depolymerization reagent CytoD (0.5 µM, N_{exp} = 2 & N_{cell} = 66). (C) EGFP-wt-CFTR entry into platforms after Thaps (N_{exp} = 8 & N_{cell} = 132) was not affected by CytoD (N_{exp} = 2 & N_{cell} = 48), indicating the actin cytoskeleton does not influence CFTR cluster or platform formations at the PM. (D & E) HBE cells were transduced with an adenovirus containing 3HA-wt-CFTR, treated or not with 2BP (10 µM for 24 h) then immuno-stained with HA antibody. General inhibition of palmitoylation (E) did not alter CFTR cluster formation at the PM, strongly indicating the lack of role for palmitoylation in this process.

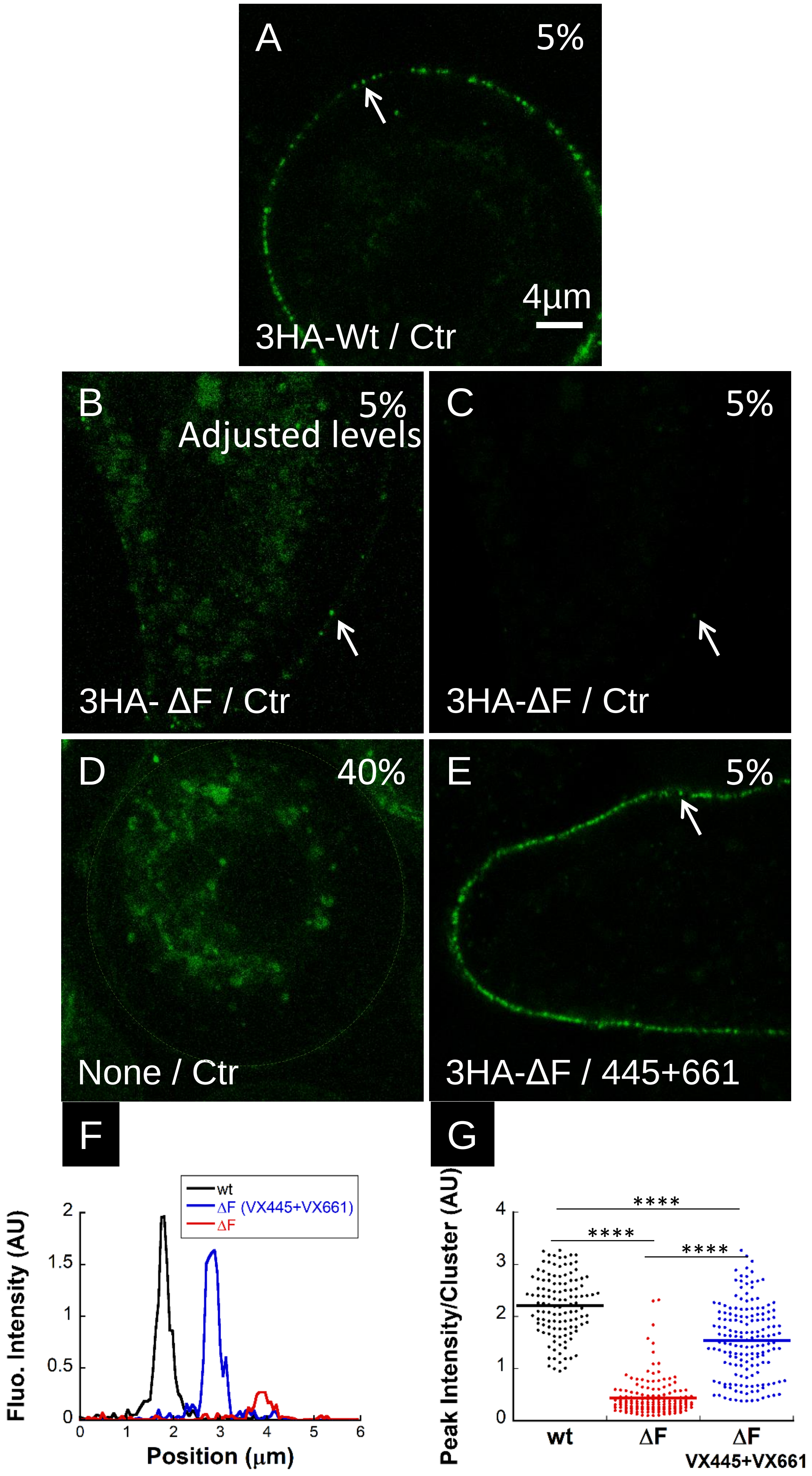

Journal of Cell Science • Supplementary information

Fig. S5. F508del-CFTR is expressed at the PM. CF HBE cells were transduced with adenoviruses containing 3HA-wt- or 3HA-F508del-CFTR, exposed to anti-HA antibody and fixed. (A) Plasma membrane distribution of wt-CFTR and cluster formation (white arrow, N_{exp} = 5 & N_{cell} = 115). (B) Under the same imaging conditions (5% laser power), some F508del-CFTR is trafficked to the plasma membrane and forms dimmer (5-fold) less abundant (4-fold) puncta (white arrow, N_{exp} = 5 & N_{cell} = 130) compared to wt-CFTR. (C) Highlighting the dim F508del-CFTR puncta by adjusting the fluorescence intensity levels of (B). (D) Only intracellular background fluorescence is detected using high laser power (40%) in cells that do not express CFTR. (E) Trikafta correctors (VX-445 + VX-661, 24 h) restore F508del-CFTR membrane expression and cluster formation, brightness and abundance (white arrow, N_{exp} = 5 & N_{cell} = 145). (F) Representative fluorescence Intensity profile of individual wt-CFTR cluster and a F508del-CFTR punctum (before and after Trikafta correction). (G) Collective peak fluorescence analysis reveals a significant difference in mean fluorescence intensity of wt-CFTR and F508del-CFTR (I_{wt} = 2.21±0.05 arbitrary units (n_{cell} = 4, $n_{cluster}$ = 118), & $I_{\Delta F}$ = 0.44 ± 0.03 arbitrary units (n_{cell} = 20 cells, $n_{cluster}$ = 156)), and the restoration of F508del-CFTR clustering after correction ($I_{\Delta F(VX)}$= 1.54 ± 0.06 arbitrary units (n_{cell} = 5, $n_{cluster}$ = 162)). The experiment was repeated 5 times. Errors are s.e.m. ****$p<0.0005$.

Journal of Cell Science • Supplementary information

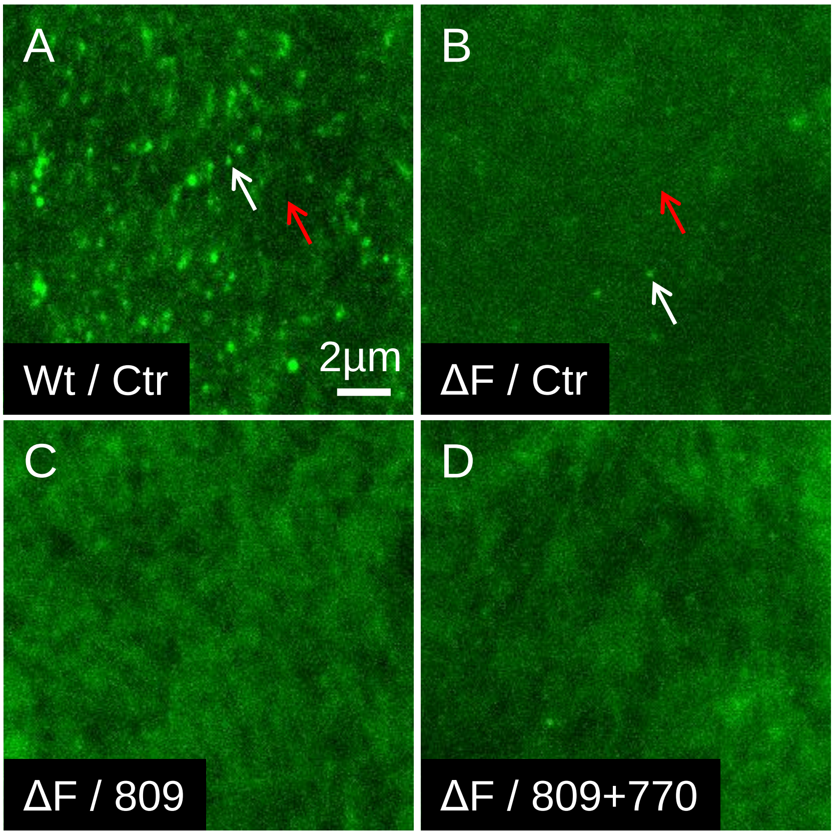

Fig. S6. Lumacaftor and Ivacaftor do not restore F508el-CFTR clustering. HBE cells transduced with EGFP-wt-CFTR or EGFP-F508del-CFTR adenoviruses were treated or not with lumacaftor (VX809, 1 µM) or in combination with Ivacaftor (VX770, 100 nM) for 24 h. (A) The plasma membrane distribution of wt-CFTR under Ctr conditions shows clustering (white arrow). (B) F508del-CFTR distribution is diffuse (red arrow) and lacks clustering except for small, sparce and dim puncta. (C) Lumacaftor correction does not restore F508del-CFTR clustering. (D) The combination of lumacaftor and Ivacaftor treatment also does not restore F508del-CFTR cluster formation at the plasma membrane.

Journal of Cell Science • Supplementary information

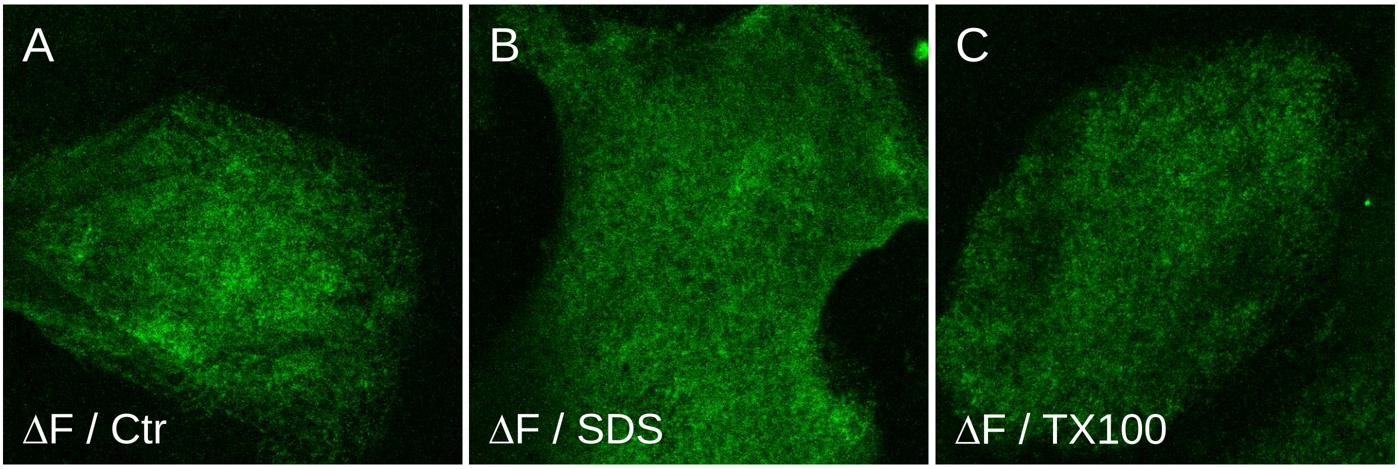

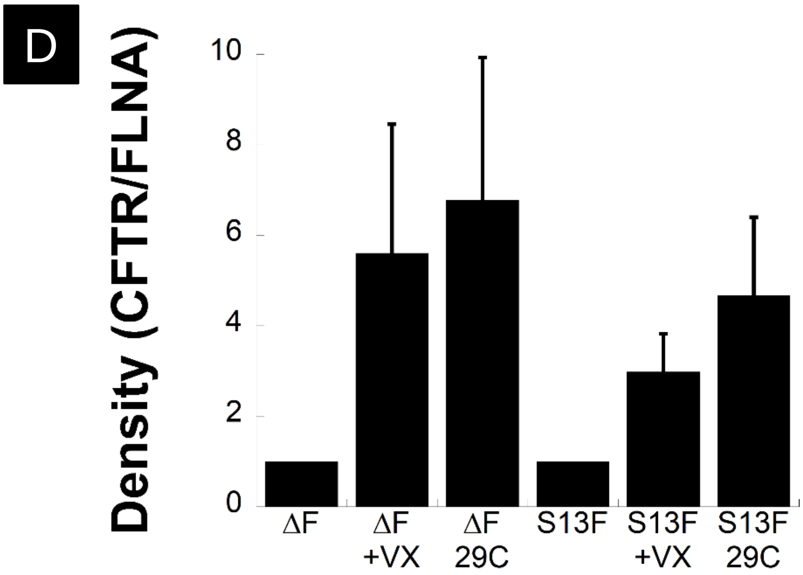

Fig. S7. (A – C) No phase separation of F508del-CFTR is observed following a treatment with detergents suggesting lack of clustering due to its exclusion from lipid rafts. (D) **: Band density quantification**. Comparing the mature F508del-CFTR and S13F-CFTR band density (normalized to FLNA) under Ctr, VX or low temp (29 ºC) conditions. VX445 + VX661 correction increased the band density by 6-fold for F508del-CFTR and by 3-fold for S13F-CFTR. $N_{exp} = 3$, $p < 0.05$ when comparing to its corresponding control.

Journal of Cell Science • Supplementary information

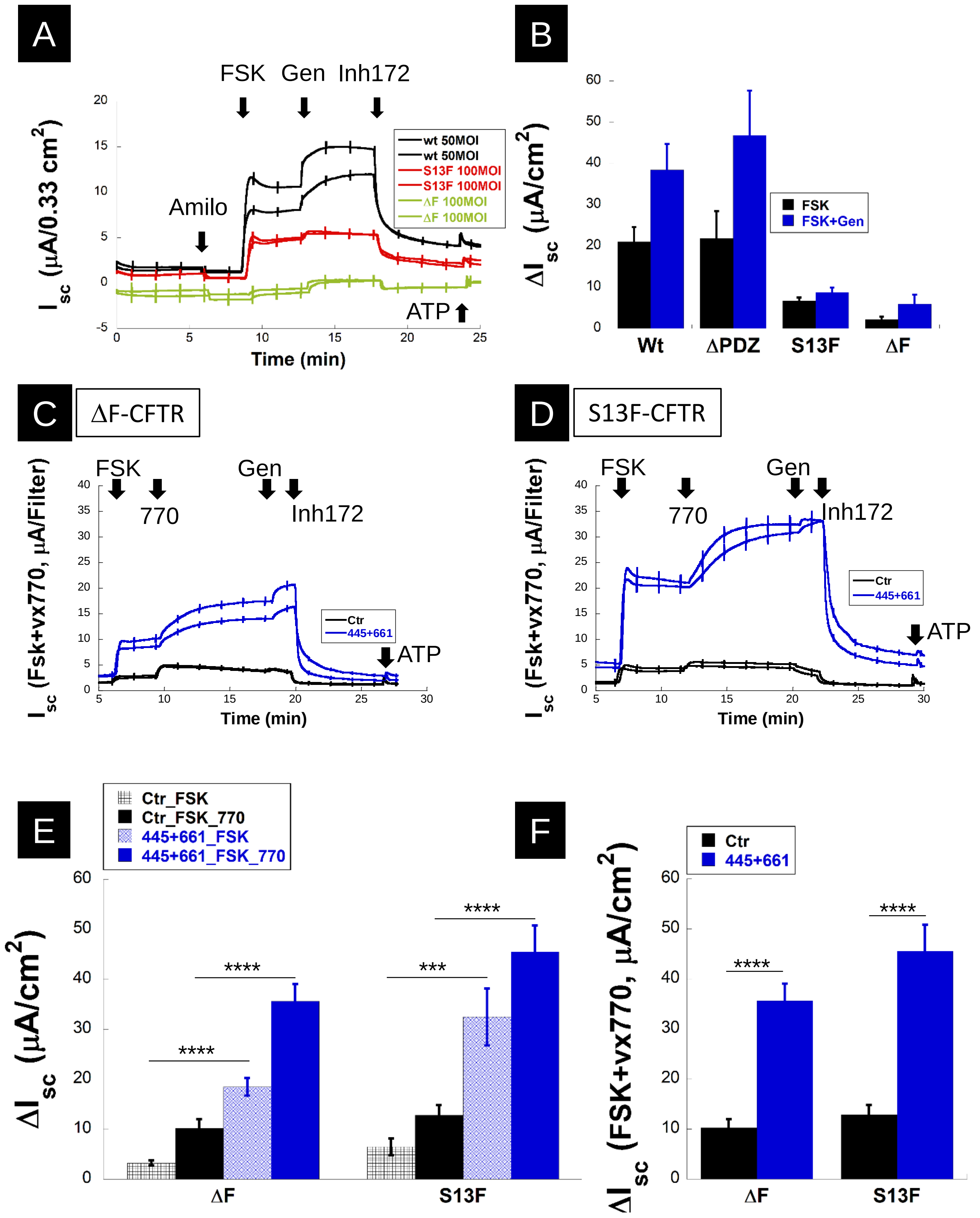

Journal of Cell Science • Supplementary information

Fig. S8. Trikafta correctors rescue F508del-CFTR and S13F-CFTR channel activity. CF HBE cells were transduced with wt-, F508del-, ΔPDZ- or S13F-CFTR adenoviruses at seeding then maintained for 4 days under submerged conditions and for 7 days under air-liquid-interface (ALI) conditions. (A) Representative short circuit current (I_{sc}) traces for wt-, F508del and S13F-CFTR. (B) At similar expression levels, the largest I_{sc} stimulation by forskolin + genistein is observed with wt-CFTR, then with S13F-CFTR while F508del-CFTR is least responsive. (C & D) Comparison of I_{sc} traces for F508del-CFTR and S13F-CFTR before (black) and after VX-445 + VX-661 correction (blue) (3 µM each, 24h). (E & F) Under our experimental conditions and before correction, both F508del- and S13F-CFTR show similar functional behavior in response to FSK and 100 nM VX-770VX-770. (F) VX-445 + VX-661 correction increases the total functional response (FSK+770) of both F508del- and S13F-CFTR by 3.5- & 4.5-fold, respectively. (n = 6 filters /condition, N_{exp} = 3). *** p< 0.0025, **** p<0.0005. Errors are s.e.m.

Journal of Cell Science • Supplementary information
