# Supplementary material for: Lipid-driven CFTR clustering is impaired in cystic fibrosis and restored by corrector drugs
Source: J Cell Sci. 2022 Mar 7;135(5):jcs259002. doi: 10.1242/jcs.259002 (PMC8976878; doi:10.1242/jcs.259002)
Supplement: Reviewer comments [file joces-135-259002_review_history.pdf]

Lipid-driven CFTR clustering is impaired in cystic fibrosis and restored by corrector drugs

Asmahan Abu-Arish, Elvis Pandžić, Yishan Luo, Yukiko Sato, Mark J. Turner, Paul W. Wiseman and John W. Hanrahan
DOI: 10.1242/jcs.259002

Editor: James Olzmann

Review timeline

Original submission:	9 June 2021
Editorial decision:	16 August 2021
First revision received:	22 November 2021
Accepted:	8 January 2022

Original submission

First decision letter

MS ID#: JOCES/2021/259002

MS TITLE: Lipid-driven CFTR clustering is impaired in CF and restored by corrector drugs

AUTHORS: Asmahan Abu-Arish, Elvis Pandzic, Yishan Luo, Yukiko Sato, Mark John Turner, Paul W Wiseman, and John W. Hanrahan
ARTICLE TYPE: Research Article

We have now reached a decision on the above manuscript.

To see the reviewers' reports and a copy of this decision letter, please go to: https://submit-jcs.biologists.org and click on the 'Manuscripts with Decisions' queue in the Author Area. (Corresponding author only has access to reviews.)

As you will see, the reviewers raise a number of substantial criticisms, several requiring additional experimentation to address. These concerns prevent me from accepting the paper at this stage. They suggest, however, that a revised version might prove acceptable, if you can address their concerns. If you think that you can deal satisfactorily with the criticisms on revision, I would be willing to consider a revised manuscript. We would then return it to the reviewers.

We are aware that you may be experiencing disruption to the normal running of your lab that makes experimental revisions challenging. If it would be helpful, we encourage you to contact us to discuss your revision in greater detail. Please send us a point-by-point response indicating where you are able to address concerns raised (either experimentally or by changes to the text) and where you will not be able to do so within the normal timeframe of a revision. We will then provide further guidance. Please also note that we are happy to extend revision timeframes as necessary.

Please ensure that you clearly highlight all changes made in the revised manuscript. Please avoid using 'Tracked changes' in Word files as these are lost in PDF conversion.

I should be grateful if you would also provide a point-by-point response detailing how you have dealt with the points raised by the reviewers in the 'Response to Reviewers' box. Please attend to

© 2022. Published by The Company of Biologists under the terms of the Creative Commons Attribution License (https://creativecommons.org/licenses/by/4.0/).

all of the reviewers' comments. If you do not agree with any of their criticisms or suggestions please explain clearly why this is so.

Reviewer 1

Advance summary and potential significance to field

It has been shown before that CFTR resides in membrane rafts. This paper clarifies that protein interactions and cytoskeletal dynamics are not necessary for CFTR clustering and identifies that the effect is strictly lipid dependent. It is not clear if this mechanistic information is sufficient for the journal guidelines to justify publication. The paper also shows that CFTR modulator therapy is able to restore the clustering which has clinical significance.

Comments for the author

The manuscript by Abu-Arish et al presents an interesting and systematic approach to examining CFTR clustering in lipid rafts. The authors effectively demonstrate that CFTR clustering at the plasma membrane is lipid dependent and largely independent of protein interactions and cytoskeletal contributions. It is also demonstrated that CFTR containing trafficking mutations fail to form clusters and that effective modulator treatment corrects that phenotype.

Comments
1. Lipid content – the authors show through fluorescently labeled analogs of cholesterol and sphingomyelin the composition of CFTR membrane clusters. Given the conclusion that CFTR clustering is strictly lipid dependent, it would be of note to deplete membranes of cholesterol with cyclodextrin and determine if clustering is prevented. Presumably it would be, but a direct test of at least the cholesterol dependency on CFTR clustering would be informative. Given the fact pointed out in the discussion that cholesterol levels are reduced in rafts once sphingomyelin is cleaved to ceramide, it would be worthwhile to test directly the role of cholesterol in this process. ASM inhibition studies in the paper nicely show the SM/cer dependence.
2. VX-445/VX-661 effectively restore F508del and S13F CFTR clustering. Since G551D clusters normally, the restoration is clearly due to folding correction and/or protein density at the membrane. Is it possible to measure turnover rates of membrane CFTR from the clusters using these assays?

Minor comments
1. CD ratio is not described in the results text and the legend only states that this is density. A clearer description in the text would be helpful.
2. Some key data are relegated to supplemental data such as the amitriptyline data. Bringing some of these key experiments to the main text would help emphasize these key points.
3. It appears that some studies have very low n’s for expressing cells. For example, figure 4 only has Nexp=2 for detergent treatments.

Overall, this manuscript is well written and presents clearly that CFTR clustering is lipid dependent with other mechanisms systematically eliminated.

Reviewer 2

Advance summary and potential significance to field

CFTR is an anion channel that plays key roles in physiology and is mutated in cystic fibrosis. The authors previously found that CFTR expressed in HBE cells is diffuse and localized to clusters, potentially liquid ordered domains. Here they examine the mechanism of CFTR clustering, examining interactions with the cytoskeleton, the contribution of CFTR palmitoylation, and the impact of diseases-associated mutations. Their data suggest that clustering is independent of cytoskeleton interactions and palmitoylation, but can be driven by lipid order. They also observe impaired clustering by disease-associated mutants that can be rescued by corrector drugs. While the observations are potentially interesting, lack of controls and quantification in several figures is concerning and must be addressed prior to publication.

© 2022. Published by The Company of Biologists under the terms of the Creative Commons Attribution License (https://creativecommons.org/licenses/by/4.0/).

Comments for the author

Comment 1: In figure 1, several controls should be added. What is the distribution of WT-CFTR in the absence of cholesterol and C12-SM? Is it still localized to these clusters? What about a control of C12-SM alone without thapsigargin added? Does C12-SM induce these larger aggregates or would C12-SM and CFTR be present in distinct subdomains? Quantification of these clusters and the colocalization would be helpful for comparison. The imaging method should also be described in more detail – how do we know these are plasma membrane clusters and not altered ER structures? Markers? TIRF?
Comment 2: While the effects of thapsigargin are striking, it is unclear why thapsigargin was employed when it is so artificial, inducing large calcium intracellular calcium concentrations for extended periods and causing ER stress.
Could the effects be due to ER stress? Would similar changes be observed with tunicamycin for example? Are there more physiological Ca signaling mechanisms that induce similar changes in CFTR localization?
Comment 3: Western blotting should be performed to allow comparison of mutant CFTR expression levels and trafficking. For example, the deltaPDZ mutant in Figure 2. Western blotting should also be used to confirm RNAi depletion of FLNA in Figure 2 since mRNA levels do not always reflect protein depletion.
Comment 4: In order to make conclusions about CFTR palmitoylation, the status of CFTR palmitoylation must be measured. Simply citing a reference is insufficient.
Is CFTR palmitoylated in these cells and does C1395A affect its palmitoylation?
In the figure panel, the mutant should be labeled as C1395A not Palm (this was confusing). Why was C524 not considered? The distance from the membrane was mentioned, but is this based upon structures where C524 is not palmitoylated? If C524 is palmitoylated this could result in structural rearrangement and tethering to the membrane. It is premature to conclude that palmitoylation is not involved based upon the data presented.
Comment 5: Markers of lipid order and disorder are required for Figure 4 where detergents are used to induce lipid phase separation. Do these structures contain cholesterol and SM similar to the observed CFTR containing domains observed under other conditions?
Comment 6: It is unclear whether the F508 mutant fails to cluster due to difference in structure or differences in the amount present on the plasma membrane. The correctors affect both?
Comment 7: The clusters indicated with the white arrow in Fig 5g are not obvious. A marker would be helpful (cholesterol?).
Comment 8: In figure 7g, FLNA is immunoprecipitated and more S13F co-immunoprecipitates in cells treated with correctors or incubated under low temperature. However, considerably less S13F was IP'd in the control condition.
Perhaps 5-fold less? This experiment must be performed with equivalent amounts of S13F IP'd. Since this was performed 3x, quantification of CFTR could be included and normalized to the IP'd S13F.
Comment 9: Why was cholera toxin used in the final figure? Would similar results be observed with cholesterol and SM?

First revision

Author response to reviewers' comments

Responses to Reviewer 1
We thank the reviewers for their comments. Addressing them have significantly improved the manuscript.

Comments

1. Lipid content – the authors show through fluorescently labeled analogs of cholesterol and sphingomyelin the composition of CFTR membrane clusters. Given the conclusion that CFTR clustering is strictly lipid dependent, it would be of note to deplete membranes of cholesterol with cyclodextrin and determine if clustering is prevented. Presumably it would be, but a direct test of at least the cholesterol dependency on CFTR clustering would be informative.

© 2022. Published by The Company of Biologists under the terms of the Creative Commons Attribution License (https://creativecommons.org/licenses/by/4.0/).

Response: The reviewer is correct to point that out, and this sort of experiment was performed in our 2015 Biophysical Journal paper (Abu-Arish et al., 2015). The enzyme cholesterol oxidase (COase), which gently depletes the plasma membrane of cholesterol reduced CFTR clustering. In that study we quantified the effect of cholesterol depletion on CFTR cluster size (degree of aggregation) using image correlation spectroscopy (ICS) analysis, and on CFTR mobility at the plasma membrane using k-space ICS (kICS). We also increased cholesterol concentration in the plasma membrane using cholesterol esterase (CEase). Whereas cholesterol depletion reduced cluster size and increased CFTR mobility, cholesterol supplementation increased CFTR cluster size and confinement, suggesting that cholesterol is essential for CFTR clustering at the plasma membrane.
We used an enzymatic method to deplete cholesterol because it is less harsh than methyl-ß-cyclodextrin (MßCD). Nevertheless, we performed the following experiment with MßCD to address the reviewer's concern. Below are two confocal images of EGFP- wt-CFTR at the plasma membrane of HBE cells under control conditions (Ctr, A) and after treatment with MßCD (3.8 mM, B). Cholesterol depletion caused the loss of CFTR clusters (white arrow) and peripheral localization (yellow arrow). Artifactual distributions of CFTR was induced by the reagent in some MßCD-treated cells (Data not shown).

***NOTE**: We have removed unpublished data that had been provided for the referees in confidence.*

– Given the fact pointed out in the discussion that cholesterol levels are reduced in rafts once sphingomyelin is cleaved to ceramide it would be worthwhile to test directly the role of cholesterol in this process. ASM inhibition studies in the paper nicely show the SM/cer dependence.

Response: We have previously shown that cholesterol and intact lipid rafts are essential for the formation of ceramide-rich platforms (see Figures 6 & 7 in (Abu-Arish et al., 2019)). The physiological agonist VIP triggers the formation of ceramide-rich platforms containing CFTR whereas depleting cholesterol prior to VIP treatment prevents formation of the platforms indicating that cholesterol is essential for platform formation as expected. Whether cholesterol remains inside the platforms or is displaced by ceramide is uncertain and may vary with cell type. Ceramide generation in the detergent-resistant fraction from S16 cells membranes reduces cholesterol concentration by 25% in that fraction after 2 h treatment with bacterial SMase (Yu et al., 2005). Those authors concluded that ceramide generation in microdomains displaces cholesterol, but whether that occurs in our system is unknown.
When we label cells with Bodipy-cholesterol using MßCD as a vehicle for fast incorporation (see B) and then trigger platform formation using thapsigargin or SDS (see C & D), both agents increase Bodipy fluorescence in platforms even at low cholesterol concentrations ((C– E), blue arrows). Furthermore, Bodipy-Cholesterol platforms colocalized spatially with mCherry-wt-CFTR-containing platforms (see E – G), and since C12-SM in the present study was found enriched in platforms containing mCherry-wt-CFTR (main manuscript Figure 1D – F), this indicates that cholesterol is enriched inside ceramide-rich platforms. We hope to plan to study this enrichment in the future.

***NOTE**: We have removed unpublished data that had been provided for the referees in confidence.*

***NOTE**: We have removed unpublished data that had been provided for the referees in confidence.*

© 2022. Published by The Company of Biologists under the terms of the Creative Commons Attribution License (https://creativecommons.org/licenses/by/4.0/).

2. VX-445/VX-661 effectively restore F508del and S13F CFTR clustering. Since G551D clusters normally, the restoration is clearly due to folding correction and/or protein density at the membrane. Is it possible to measure turnover rates of membrane CFTR from the clusters using these assays?

Response: We have performed an experiment to address this comment and measure wt-CFTR turnover from within membrane clusters. Using WT-CFTR that has a 3HA-tag inserted in the 4th extracellular loop, we immunolabeled cells extracellularly as described in Fig. S6 using an anti-HA antibody at 4°C (without cell permeabilization). Cells were then washed and warmed to 37 °C under Ctr conditions (A&B) or with MβCD treatment (C&D, to disrupt CFTR clustering inside lipid rafts) for 0 or 2 hrs. Cells were then fixed, permeabilized and labeled with a secondary antibody. The intracellular-to-membrane fluorescence intensity ratio of labeled CFTR was determined to estimate CFTR turn over and assess the role of CFTR clustering on its membrane stability.

***NOTE**: We have removed unpublished data that had been provided for the referees in confidence.*

The ratio of intracellular-to-membrane fluorescence measured at 0 h was very low under both Ctr and MβCD conditions as expected. CFTR internalization caused a marked reduction in CFTR membrane staining as shown (panels B&D) with the largest reduction occurring after exposure to MβCD for 2 hrs (D). This implicates membrane cholesterol and lipid rafts in membrane CFTR stability. The increase in intracellular CFTR after 2 h at 37 °C demonstrates internalization of the extracellularly labelled CFTR, with the largest increase occurring after MβCD treatment (D). As shown in panel E, cells treated with MβCD for 2 hrs showed a 5-fold increase in CFTR internalization relative to Ctr cells, further evidence that CFTR clusters in cholesterol-dependent microdomains influence its membrane stability.
We would be happy to add these important results to the main manuscript however there is a limit on the number of figures allowed by the journal. We would appreciate guidance from the reviewer where this figure might be inserted. The experiment was repeated twice, with Ncell: nctr(0hr)=35, nctr(2hr)=35, nMβCD(0hr)=45, nMβCD(2hr)=50. The difference between Ctr and MβCD was significant ($p<0.0005$) at 2 h according to an unpaired t-test.

Minor comments

1. CD ratio is not described in the results text and the legend only states that this is density. A clearer description in the text would be helpful.

   Response: The cluster density (CS) was defined in the methods section but now is also clarified in the text and the figure legend.

2. Some key data are relegated to supplemental data such as the amitriptyline data. Bringing some of these key experiments to the main text would help emphasize these key points.

   Response: We have exchanged Figure S4(A-D) and Figure 3(D-F) to restore these data to the main manuscript. We were forced to relegate this data to supplemental data due to the restriction on the number of figures allowed in the main manuscript.

3. It appears that some studies have very low n's for expressing cells. For example, figure 4 only has Nexp=2 for detergent treatments.

   Response: We have performed more experiments as shown in the legends of Figure 3(G&H) and Figure 4(A-I). As indicated throughout the manuscript, we report both the number of independent experiments Nexp and the number of cells analyzed ncells, which are viewed as semi-independent technical replicates.

© 2022. Published by The Company of Biologists under the terms of the Creative Commons Attribution License (https://creativecommons.org/licenses/by/4.0/).

Figure 3(G & H) legend:
Ctr conditions are now (Nexp = 4 & Ncell = 114) Thaps treatment are now (Nexp = 3 & Ncell = 69) Figure 4(A - I) legend:
A7 cells: are now (SDS: Nexp = 6 & Ncell = 281, TX100: Nexp = 3 & Ncell = 112) M2 cells: are now (SDS: Nexp = 4 & Ncell = 123, TX100: Nexp = 3 & Ncell = 83) HBE cells: are now (SDS: Nexp = 5 & Ncell = 187, TX100: Nexp = 4 & Ncell = 138

Reviewer 2 Comments for the Author...

Comments:

1. In figure 1, several controls should be added. What is the distribution of WT-CFTR in the absence of cholesterol and C12-SM? Is it still localized to these clusters?

Response: In the absence of exogenous Bodipy-cholesterol or Bodipy-C12-SM, WT-CFTR forms clusters as described in our previous publications (Abu-Arish et al., 2015, Abu-Arish et al., 2019). We showed that some CFTR is distributed inside clusters at the plasma membrane of HBE cells and we quantified that localization (Abu-Arish et al., 2015). The importance of endogenous cholesterol in CFTR clustering was revealed by depleting it with the enzyme cholesterol oxidase or by supplementing it using the enzyme cholesterol esterase. CFTR clustering in the absence of exogenous cholesterol or sphingomyelin was also confirmed throughout the current manuscript (e.g. Figure 2A, Figure 3A & D, Figure 4G, Figure 5A, etc). We would have liked to add a confocal image showing CFTR clusters under control conditions in Figure 1, however due to limited space we put the controls in later figures. Please see the example below (and those in Figs 2A, 3A, 3H).

***NOTE**: We have removed unpublished data that had been provided for the referees in confidence.*

- What about a control of C12-SM alone without thapsigargin added? Does C12-SM induce these larger aggregates or would C12-SM and CFTR be present in distinct subdomains?

Response: This has now been added as figure 1H & 1I (see below). Figure 1H highlights the distribution of C12-SM at the plasma membrane of HBE cells under control conditions. No platform formation was seen prior to thapsigargin treatment demonstrating that C12-SM on its own does not induce platform formation. In fact, C12-SM under Ctr conditions showed a rather even distribution through the plasma membrane and with a distinct distribution at the cell rim. This distribution is not surprising as 70% of membrane lipids are SM and are distributed throughout the membrane. Figure 1I shows the formation of C12-SM-rich platforms shortly after thapsigargin treatment and in the absence of CFTR. Very high spatial colocalization between C12-SM and CFTR is evident inside platforms, Figure 1F.

***NOTE**: We have removed unpublished data that had been provided for the referees in confidence.*

- Quantification of these clusters and the colocalization would be helpful for comparison.

Response: Quantification of the CFTR/cholesterol colocalization in clusters and CFTR/C12- SM colocalization in platforms has been added to the main manuscript (Fig. 1G, see below). Mander’s overlap coefficient was used because it is a purely spatial parameter that is insensitive to differences in fluorescence intensity between the 2 channels. Mander’s coefficient was calculated using the ZEN software. Spatial colocalizations between CFTR and Bodipy-cholesterol and CFTR and Bodipy-C12-SM were ~90%.

© 2022. Published by The Company of Biologists under the terms of the Creative Commons Attribution License (https://creativecommons.org/licenses/by/4.0/).

NOTE: *We have removed unpublished data that had been provided for the referees in confidence.*

- The imaging method should also be described in more detail – how do we know these are plasma membrane clusters and not altered ER structures? Markers? TIRF?

Response: This is an important question. Using a WT-CFTR construct with a 3HA-tag inserted at the 4th extracellular loop together with immune-staining maneuver we show in Figure S6 that WT-CFTR is expressed at the PM, and it forms clusters (bead-like). Comparing the brightness of WT-CFTR clusters (Fig. S6A) with F508del-CFTR puncta (Fig. S6C) reveals that WT-CFTR clusters are 5x brighter, suggesting there are ~5 CFTR per cluster. Cluster profiles are shown in Fig. S6F, and their quantification in Fig. S5G. This extracellular tagging method excludes any ER contribution within the timeframe of the experiment. Also, using the same method, the images below strictly show membrane CFTR clusters (white arrow) and platforms (blue arrow) without any contribution from ER-CFTR population.

NOTE: *We have removed unpublished data that had been provided for the referees in confidence.*

2. While the effects of thapsigargin are striking, it is unclear why thapsigargin was employed when it is so artificial, inducing large calcium intracellular calcium concentrations for extended periods and causing ER stress. Could the effects be due to ER stress? Would similar changes be observed with tunicamycin for example? Are there more physiological Ca signaling mechanisms that induce similar changes in CFTR localization?

Response: We previously utilized two physiological agonists that mobilize calcium, vasoactive intestinal peptide (VIP) and carbachol. They both triggered the formation of ceramide-rich platforms at the plasma membrane and CFTR incorporation inside the platforms (Abu-Arish et al., 2019). We used thapsigargin to raise calcium rather than a receptor agonist because most receptors activate multiple signalling pathways. While thapsigargin is not physiological, it is reproducible, less toxic than calcium ionophores, and works similarly on diverse cell types.

3. Western blotting should be performed to allow comparison of mutant CFTR expression levels and trafficking. For example, the deltaPDZ mutant in Figure 2.

Response: We have expressed WT-CFTR and the truncated PDZdel-CFTR (clone 61) in HBE cells and performed co-immunoprecipitations to validate their trafficking and interaction with the PDZ-protein NHERF1. Both WT- and PDZdel-CFTR (clone 61, red arrow) are highly expressed as C-band, indicating they traffic through the Golgi and probably reach the plasma membrane. As shown below, WT-CFTR interacts with NHERF1 whereas PDZdel-CFTR does not (yellow arrow). For comparison, lane C indicates control cells that do not express CFTR.

Furthermore, expression and trafficking of EGFP-tagged WT-CFTR, PDZdel-CFTR, C1395A- CFTR and G551D-CFTR that formed clusters were confirmed to have high expression (in the main manuscript). Comparing their expression profiles and membrane distributions to those of F508del-CFTR and S13F-CFTR indicates that the PDZdel mutation does not affect CFTR expression or trafficking.

NOTE: *We have removed unpublished data that had been provided for the referees in confidence.*

- Western blotting should also be used to confirm RNAi depletion of FLNA in Figure 2 since mRNA levels do not always reflect protein depletion.

Response: We confirmed protein depletion using quantitative immunostaining, which revealed a large decrease (80%) in Filamin A immunofluorescence after siRNA knockdown (compare scrambled control Scr, Fig. S2A and dicer 13.2 in Fig. S2B). 20 ROI were used for the Scr control and 50 ROI for the 13.2 dicer. Each ROI contained 2 – 3 cells.

© 2022. Published by The Company of Biologists under the terms of the Creative Commons Attribution License (https://creativecommons.org/licenses/by/4.0/).

***NOTE**: We have removed unpublished data that had been provided for the referees in confidence.*

4. In order to make conclusions about CFTR palmitoylation, the status of CFTR palmitoylation must be measured. Simply citing a reference is insufficient. Is CFTR palmitoylated in these cells and does C1395A affect its palmitoylation? In the figure panel, the mutant should be labeled as C1395A not Palm (this was confusing). Why was C524 not considered? The distance from the membrane was mentioned, but is this based upon structures where C524 is not palmitoylated? If C524 is palmitoylated this could result in structural rearrangement and tethering to the membrane. It is premature to conclude that palmitoylation is not involved based upon the data presented.

Response: We have reworded our conclusion to be more specific, i.e. that a putative palmitoylation site C1395 does not mediate CFTR partitioning into microdomains. We have also tested the general palmitoylation inhibitor 2-bromo-palmitoyl (2BP), which should inhibit palmitoylation at all sites. 2BP did not affect clustering, providing further support for our conclusion. Using HBE cells that express the 3HA-wt-CFTR, immune-staining membrane CFTR after treatment with the inhibitor 2BP at 5 or 10 μM (higher concentrations were toxic) and for different times (2, 5 & 24 hr), did not alter wt-CFTR clustering (as shown below using 10 μM for 24h). This suggests that CFTR palmitoylation is not important for its cluster formation regardless of its position along the protein sequence. We did not repeat studies published by another group on wild type CFTR as that would require different methods (mass spectrometry rather than imaging) and would be beyond the scope of this work. Also, we would not be able to prove the null hypothesis (i.e. if we did not detect palmitoylation in the mutant, we would still not be able to conclude that it had been eliminated).

***NOTE**: We have removed unpublished data that had been provided for the referees in confidence.*

5. Markers of lipid order and disorder are required for Figure 4 where detergents are used to induce lipid phase separation. Do these structures contain cholesterol and SM similar to the observed CFTR containing domains observed under other conditions?

Response: Additional experiments have been performed using HBE and A7 cells to address this concern (see below). In HBE cells, SDS treatment triggered aggregation of Bodipy- cholesterol into platforms (B & C). Importantly, the platforms were also enriched in CFTR during stimulation with Thaps (D – F) or with SDS (G – I). This confirms that platforms triggered by SDS and thapsigargin are of similar nature.

***NOTE**: We have removed unpublished data that had been provided for the referees in confidence.*

SDS treatment triggers Bodipy-C12-SM (B) and Bodipy-Chol (D) aggregation into platforms in A7 cells (which normally lack clusters), and that these platforms are also enriched with CFTR. (see below)

***NOTE**: We have removed unpublished data that had been provided for the referees in confidence.*

6. It is unclear whether the F508 mutant fails to cluster due to difference in structure or differences in the amount present on the plasma membrane. The correctors affect both?

Response: We agree that it is difficult to differentiate the effect of folding correction on F508del-CFTR trafficking and its interactions with lipids in the plasma membrane. Although partial correction by Trikafta probably affects both, the aggregation and mobility studies in Figure 6 (A&B) support some effect of the mutations on CFTR-lipid interactions. After correction of F508del-CFTR, clusters were consistently smaller and more mobile compared to WT-CFTR suggesting a different lipid environment. That difference was sustained even after 3 days exposure to Trikafta. It seems

© 2022. Published by The Company of Biologists under the terms of the Creative Commons Attribution License (https://creativecommons.org/licenses/by/4.0/).

that abundance is not the only determinant of CFTR clustering and confinement. Trikafta does not fully correct CFTR folding and therefore doesn't fully restore its trafficking or its clustering in lipid microdomains.

7. The clusters indicated with the white arrow in Fig 5g are not obvious. A marker would be helpful (cholesterol?).

Response: We have added an inset (Fig. 5G & G', see below) to highlight F508del-CFTR clustering after Trikafta correction.

***NOTE**: We have removed unpublished data that had been provided for the referees in confidence.*

We do not have an mCherry-F508del-CFTR construct for colocalization with Bodipy- cholesterol and generally try to avoid using mCherry-tagged proteins due to their tendency to aggregate artifactually. (See below for a comparison of mCherry-WT-CFTR and EGFP-WT- CFTR under control and thapsigargin treatment conditions. Note the intracellular aggregates of mCherry-WT-CFTR that obscure platforms at the cell surface compared to the clearly visible clusters and platforms containing EGFP-WT-CFTR).

***NOTE**: We have removed unpublished data that had been provided for the referees in confidence.*

- In figure 7g, FLNA is immunoprecipitated and more S13F co- immunoprecipitates in cells treated with correctors or incubated under low temperature. However, considerably less S13F was IP'd in the control condition. Perhaps 5-fold less? This experiment must be performed with equivalent amounts of S13F IP'd. Since this was performed 3x, quantification of CFTR could be included and normalized to the IP'd S13F.

Response: The quantification was performed and added as Figure S9. There is variation between experiments as indicated by the standard error. The increase in co- immunoprecipitated S13F-CFTR or F508del-CFTR after correction by low temperature or Trikafta varied between 50% to 1200%, however the increase is significant at $p<0.05$ (See below).

***NOTE**: We have removed unpublished data that had been provided for the referees in confidence.*

8. Why was cholera toxin used in the final figure? Would similar results be observed with cholesterol and SM?

Response: The aim of this experiment in Figure 8 was to examine the effect of CFTR on lipid raft formation by measuring raft size without altering the plasma membrane, therefore we did not use exogenous lipids. We were concerned that adding exogenous SM or cholesterol would mask the real effect of CFTR. CF cells have many lipid abnormalities including altered cholesterol distribution (White et al., 2004, White et al., 2007) and an increased ratio of long chain to very long chain ceramides, which also suggests an altered SM profile (Garić et al., 2017). To examine the impact of CFTR on membrane lipids we therefore used the well-established lipid raft marker cholera toxin to label them without manipulating the membrane lipid profile.

© 2022. Published by The Company of Biologists under the terms of the Creative Commons Attribution License (https://creativecommons.org/licenses/by/4.0/).

References:

ABU-ARISH, A., PANDZIC, E., GOEPP, J., MATTHES, E., HANRAHAN, J. W. & WISEMAN, P. W. 2015. Cholesterol Modulates CFTR confinement in the plasma membrane of primary epithelial cells. *Biophys J*, 109, 10.

ABU-ARISH, A., PANDZIC, E., KIM, D., TSENG, H. W., WISEMAN, P. W. & HANRAHAN, J. W. 2019. Agonists that stimulate secretion promote the recruitment of CFTR inot membrane lipid microdomains. *J Gen Physiol*, 151, 16.

GARIĆ, D., DE SANCTIS, J. B., WOJEWODKA, G., HOULE, D., CUPRI, S., ABU-ARISH, A., HANRAHAN, J. W., HAJDUCH, M., MATOUK, E. & RADZIOCH, D. 2017. Fenretinide differentially modulates the levels of long- and very long-chain ceramides by downregulating Cers5 enzyme: evidence from bench to bedside. *J Mol Med (Berl)*, 95, 1053-1064.

WHITE, N. M., COREY, D. A. & KELLEY, T. J. 2004. Mechanistic similarities between cultured cell models of cystic fibrosis and niemann-pick type *C Am J Respir Cell Mol Biol*, 31, 6.

WHITE, N. M., JIANG, D., BURGESS, J. D., BEDERMAN, I. R., PREVIS, S. F. & KELLEY, T. J. 2007. Altered cholesterol homeostasis in cultured and in vivo models of cystic fibrosis. *Am J Physiol Lung Cell Mol Physiol*, 292, 11.

YU, C., ALTERMAN, M. & DOBROWSKY, R. T. 2005. Ceramide displaces cholesterol from lipid rafts and decreases the association of the cholesterol binding protein caveolin-1. *J Lipid Res*, 46, 1678-91.

Second decision letter

MS ID#: JOCES/2021/259002

MS TITLE: Lipid-driven CFTR clustering is impaired in CF and restored by corrector drugs

AUTHORS: Asmahan Abu-Arish, Elvis Pandzic, Yishan Luo, Yukiko Sato, Mark John Turner, Paul W Wiseman, and John W. Hanrahan
ARTICLE TYPE: Research Article

I am happy to tell you that your manuscript has been accepted for publication in Journal of Cell Science, pending standard ethics checks.

© 2022. Published by The Company of Biologists under the terms of the Creative Commons Attribution License (https://creativecommons.org/licenses/by/4.0/).
